# Knowledge, attitude, and practice (KAP), and acceptance and willingness to pay (WTP) for mosquito-borne diseases control through sterile mosquito release in Bangkok, Thailand

**DOI:** 10.1371/journal.pntd.0011935

**Published:** 2025-07-28

**Authors:** Pattamaporn Kittayapong, Suwannapa Ninphanomchai, Namon Jalichandra, Luechai Sringernyuang, Penchan Sherer, Natthani Meemon

**Affiliations:** 1 Center of Excellence for Vectors and Vector-Borne Diseases, Faculty of Science, Mahidol University at Salaya, Nakhon Pathom, Thailand; 2 EcoHealth Research Center, Go Green Co., Ltd., Na Muang Sub-District, Muang District, Chachoengsao, Thailand; 3 Department of Society and Health, Faculty of Social Science and Humanities, Mahidol University at Salaya, Nakhon Pathom, Thailand; Centers for Disease Control and Prevention, UNITED STATES OF AMERICA

## Abstract

**Background:**

Arboviral diseases such as dengue, chikungunya and Zika are public health concerns worldwide. Prevention and control of these diseases still depend on controlling *Aedes aegypti* mosquito vectors. Sterile insect technique (SIT) and incompatible insect technique (IIT) are environmental friendly approaches that show promising impacts. In order to plan an implementation of SIT/IIT technology, background knowledge, attitudes and practices (KAP) related to mosquito-borne diseases, mosquito vectors and their prevention and control, as well as acceptance and willingness to pay (WTP) for the technology, in the targeted communities are needed.

**Methodology/Principal findings:**

In this paper, we conducted questionnaire surveys on KAP and WTP in 400 sampled households in seven communities located in two districts in Bangkok, Thailand. Multivariate logistic regressions analysis was used to determine the association among knowledge, attitudes and practices regarding dengue, chikungunya, and Zika. Our findings indicated that participants had high knowledge on dengue (85.25%), and they were more concerned with the severity of dengue than chikungunya and Zika. Participants with ages lower than 35 years old (*p* = 0.047) and incomes higher than 5,000 THB (*p* = 0.016) had more knowledge of mosquito vectors. Moreover, 47% of respondents had positive attitude toward sterile mosquitoes and their application in vector control even though 45.5% of them had never heard about the technology. However, the majority of them were not willing to pay (52%); and if they had to pay, the maximum would be 1–2 THB (US$ 1 = ~34 THB) per sterile mosquito, since most of them expected to receive the service as public support from the government.

**Conclusions/significance:**

Our study was the first to study KAP and WTP related to SIT/IIT technology. It provided unique insights into how communities view the technology. It also suggested potential for successful implementation with proper education as well as highlighted the need for cost-sharing strategies with government subsidization for SIT/IIT deployment. Municipal officials and community health volunteers were key communication channels and targeted public education was needed, especially on under-recognized diseases like chikungunya and Zika. These findings should be useful for health authorities in planning to integrate SIT/IIT technology with traditional approaches for disease vector control and prevention.

## Author summary

A questionnaire survey was conducted in seven communities in Bangkok, Thailand to obtain baseline information on knowledge, attitudes and practices (KAP) related to mosquito-borne diseases, i.e., dengue, chikungunya and Zika, including mosquito vectors and how to control them. The questionnaire also asked about the acceptance and willingness to pay (WTP) for sterile mosquitoes used in controlling mosquito populations. Our results showed that, from a total of 400 sampling households, about 85% of participants were familiar with dengue and its mosquito vectors, as well as their prevention and control. Furthermore, participants with lower ages and higher incomes had more knowledge on mosquito vectors (*p* < 0.05). Even though the majority of participants did not have any knowledge on sterile mosquito release, they showed a positive perception about it. However, more than half of them were not willing to pay for sterile mosquitoes, since they would like to receive them as public support from the government. If they were to pay, the cost that they could afford was 1–2 THB (~US$ 0.03- 0.06) per sterile mosquito. These findings should be useful for public health authorities when planning to apply the sterile mosquito release as an alternative mosquito control approach in Bangkok, Thailand.

## Introduction

Arthropod-borne or arbovirus diseases are public-health concerns in many regions of the world, including Thailand. Important mosquito-borne diseases affecting humans in Thailand include dengue, chikungunya and Zika [1]. However, there are currently no safe and highly effective vaccines available for preventing these diseases [2,3]. *Aedes* mosquitoes are the most important arbovirus vectors, and *Ae. aegypti* is the major vector of dengue and Zika virus transmission, while *Ae. albopictus* is the major vector of chikungunya virus in Thailand [4–6]. Vector control has been the principal method of preventing vector-borne diseases. In Thailand, the control of vector-borne diseases, like elsewhere, largely has depended on vector management [7]. However, due to physiological resistance and behavioral avoidance to insecticides of the primary mosquito vectors of human diseases, persistent threats remain and continue to impose a public health burden to vulnerable populations [8].

Two environmental friendly insect control strategies, namely the radiation-based Sterile Insect Technique (SIT) and the *Wolbachia*-induced Incompatible Insect Technique (IIT), are based on a male release that aims to introduce sterility or lethality in the target population [9–11]. SIT relies on mass-rearing production, sterilization, and the recurrent release of sterile males of the target species, which are typically attained by radiation in a way that does not impair male mating and insemination capabilities. Radiation causes germ-cell chromosome fragmentation which then results in the ultimate death of the embryo [12]; hence, when wild females mate with sterile males, the eggs do not hatch [10] resulting in population suppression over the generations. An alternative is IIT, a method involving the release of *Wolbachia*-harboring insects in a natural habitat where they can mate with wild females [13,14]. IIT relies on reducing female fertility by utilizing endosymbiotic bacteria from the genus *Wolbachia*, instead of radiation, to induce a form of reproductive incompatibility known as cytoplasmic incompatibility (CI) in wild females [14]. *Wolbachia* induces a form of embryonic death [15], resulting from the sperm-egg incompatibility that occurs when *Wolbachia*-infected males mated with uninfected females or females infected with an incompatible *Wolbachia* strain. Successful implementation of SIT and IIT in field trials for mosquito control have been reported in many countries [16–19]. However, in the absence of an efficient and robust method of sex separation for *Aedes* species, it was recently suggested that irradiation (SIT) and *Wolbachia* (IIT) could be combined, which may eliminate the risks associated with the presence of a few females in sterile male batches being released in the field to suppress a target population [10,20–22].

The success of SIT or IIT programs depend largely on effective engagement and education of the community where the intervention will take place [23]. Community engagement has been the priority in SIT or IIT programs; and such programs can rapidly fail without community involvement, because community opinion and perception can positively or negatively shape the outcome of the program [24]. In our previous work, we had successfully shown proof of concept of a significant reduction of natural populations of *Ae. aegypti* mosquitoes in semi-rural settings in Plaeng Yao District, Chachoengsao Province, eastern Thailand by an application of a combined Sterile Insect Technique (SIT) and Incompatible Insect Technique (IIT) approach which relied on open field release of sterile males [21]. Currently, this combined SIT/IIT approach is in the preparation phase for a scale-up application in the capital city of Bangkok, Thailand and its implementation is estimated to launch in the middle of the year 2025.

Vector control involves collaboration with the non-health sector because it increases the sustainability of vector control programs against vector-borne diseases [25]. Knowledge, attitudes, and practices (KAP) of the populations have played a major role in the implementation of vector-borne disease control measures [26]. Understanding and enhancing households’ KAP is crucial for effective community disease control and prevention initiatives [27]. As such, KAP surveys have been conducted in many parts of the world in order to understand local contexts which can be useful for the implementation of vector control programs for dengue, chikungunya, Zika and malaria [26–33]. Some KAP surveys have used to study dengue vaccine acceptance [34], while some vector control implementers have applied KAP surveys before implementation of the new vector control technology [28].

From an economic perspective, a few studies have been done to assess the willingness to pay (WTP) for the SIT or IIT approach [35]. The WTP method has usually applied in a cost-benefit analysis and health technology assessment [36]. These are relevant methodological approaches to estimate the minimum and maximum amount (i.e., how much they are willing to pay) that an individual is willing to allocate to programs, services and health technologies [36,37]. Although many studies used the WTP approach for dengue vaccine, insecticide treated mosquito nets, and mosquito control programs [38–41], to date only one study was focused on the WTP for the SIT and it was specific only for fruit fly control [35]. Thus, the need for WTP studies with SIT/IIT mosquito control is great, especially for countries where SIT or IIT technology is planning to be implemented.

Therefore, in this study we aimed to conduct a KAP survey in order to collect information regarding community knowledge, attitudes, and perceptions about the risk of mosquito exposure and the acceptance of the new SIT/IIT technology from local residents before releasing sterile mosquitoes in a larger scale application in Bangkok, Thailand. In addition, we assessed the WTP that residents were willing to pay for sterile mosquitoes as an alternative method for mosquito control in the Thai context. Results from this study should be useful for policy makers who are considering integrating this new technology together with the routine methods for vector control in Thailand, especially in the capital city of Bangkok.

## Materials and methods

### Ethical consideration

The proposal and all relevant documents in this study were reviewed and approved by the Bangkok Metropolitan Administration Human Research Ethics Committee (BMAHREC E003q/63_EXP). Supporting letters were obtained from the District Health Offices after explaining the purpose and significance of the study. Written consents were also obtained from individual respondents from each participating household in the targeted communities.

### Study area

Administratively, Thailand is separated into three major levels: provinces, districts, and sub-districts. This study was conducted in two districts, namely Chatuchak (13°49′43″N 100°33′35″E) and Bang Khae (13°41′31″N 100°24′26″E), the eastern and western Districts in Bangkok, the capital city, located in Central Thailand with the population densities of 4,673.39 and 4,324.57 per sq.km. respectively. Geographically, Bangkok is in the Chao Phraya River delta in Thailand’s central plains. The area is flat and low-lying, with an average elevation of 1.5 m above sea level. Most of the area was originally swampland, which was gradually drained and irrigated for agriculture via the construction of canals. Bangkok was ranked as the top destination city by international visitor arrivals in the Global Destination Cities Index 2023. It is the home of approximately five million people or more than 8% of the country’s population [42]. In addition, there are a lot of people working in Bangkok, and they routinely travel to surrounding provinces in the suburbs of Bangkok, as they live outside the city center, making Bangkok an important hotspot for vector-borne diseases among both local and international visitors.

**Fig 1:**
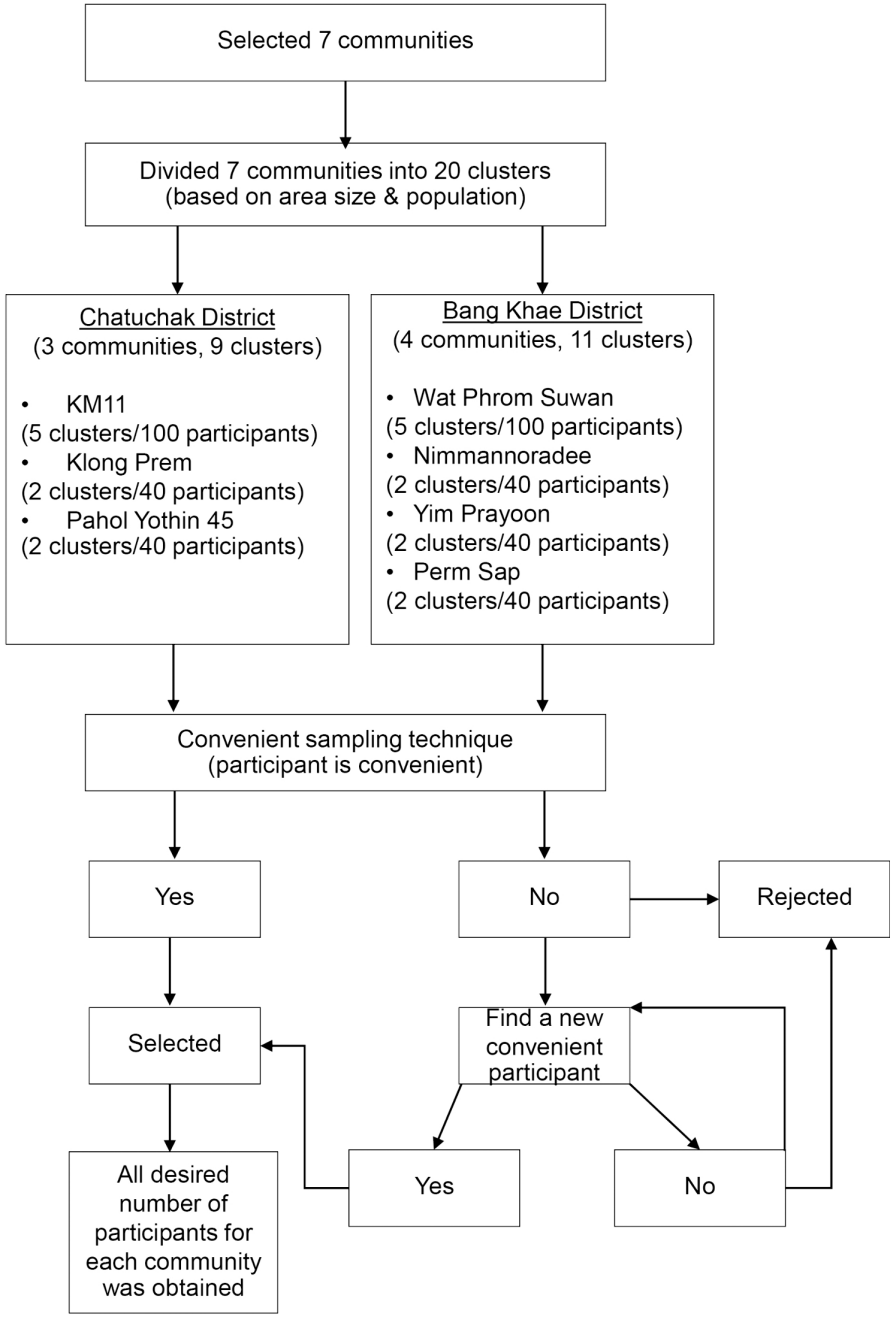
Diagram showed the sampling selection method for the questionnaire survey in Chatuchak and Bang Khae Districts, Bangkok, Thailand.

In this study, seven communities, i.e., three communities in the Chatuchak (KM11, Khlong Prem, and Pahol Yothin 45 communities) and four communities in Bang Khae (Wat Phrom Suwan, Nimmannorade, Yim Prayoon, and Perm Sap communities) districts (Fig 1), were selected based on dengue incidences from 2008-2022. Retrospective data on dengue was provided by the Health Department, Bangkok Metropolitan Administration (BMA). Briefly, Chatuchak District is more in the city center. It is a densely populated commercial service and residential area. It is the locations of four universities, twenty government offices, three popular public parks, and the famous Chatuchak weekend market where foreigners and Thai tourists come to visit and buy local souvenirs or handicraft products. Moreover, transportation in the district is mostly covered by the Bangkok Mass Transit System (BTS-sky train) and the Metropolitan Rapid Transit (MRT-underground train). Therefore, the movement of large numbers of people, both locals and foreigners, in this area is quite frequent. Bang Khae District is located in the southwest area of Bangkok. It is a mixed agricultural and residential area, with more rural areas such as agricultural plantation. Many small ports and canals are also found there. For transportation, only some parts of the district are connected to the MRT-underground train. Therefore, less frequent movement of people is found in this area.

In terms of household structures, common construction, either single or two-story houses built by woods or concrete (or sometimes connected building blocks), is found in both districts. Regarding population, people living in communities in Chatuchak District are more likely to have moved from other parts of the country or other parts of Bangkok and to live there for job opportunities, whereas those who live in communities in Bang Khae District are more likely to be local people who have stayed or lived there with their family for generations.

### Data collection

In this study, data were collected from April 2022 to August 2023 by using a constructed questionnaire. The questionnaire for the KAP survey and the willingness to pay (WTP) were adopted from instruments developed by the WHO [43], and Yeo and Shafie [39] respectively. For data collection, the research team worked in close contact with local health volunteers in each community; the community health volunteers were in charge of distribution of the questionnaire to the householders. Non-probability, purposive, and convenient sampling techniques were used to select the participants from each community (Fig 1). Community health volunteers were requested to randomly do the verbal recruitment in the community in order to find participants who were willing to answer the questionnaire. One participant per household was selected in order to reduce bias. This process was done until the desired number of participants in each community was obtained. Those who were interested in participating would primarily give a verbal consent. Then the community health volunteers would go to each participant’s household, individually distribute the questionnaire, explain how to fill it out, and also how to fill the information on the participant’s information sheet and consent form. All participants were requested to fill out and answered all questions by themselves. Also, each participant was asked to provide a written consent on the information sheet and the consent form before returning them back to the community health volunteers.

Once completed, participants would gather the completed questionnaire, together with the information sheet and the consent form. Then the community health volunteers would come and collect the questionnaire in each participant’s household. All documents from participants in the same community were gathered by the community health volunteers and sent by post to the research team for data entry and further analysis. In this study, a total sample size of 400 (n = 20 participants*20 clusters) was calculated according to the following formula [44]. This calculation was done at a confidence level of 95%, an error of less than 5%, an average dengue fever rate in Bangkok from 2014 to 2018 (P = 0.038, d = 0.09), and a drop-out rate of 15%.


$$\text{n}\;=\;[{\text{Z}}_{1-\alpha /2}^{2}\;\text{P}\;(1-\text{P})]/{\text{d}}^{2}$$

### Study instrument

A constructed questionnaire was used as a research instrument for this study. Before the questionnaire was used in the study, it was tested for internal consistency among 20 respondents during the focus group discussion between the research team and the stakeholders. The data from these participants were not included in the final analysis. The questionnaire consisted of 126 multiple-choice questions and one open question. It was mainly divided into five parts. The first part consisted of 10 questions on demographic information, such as age, gender, educational level, marital status, employment status or occupation, monthly income, number of family members, and type and characteristic of house. The second part included 29 questions on awareness and knowledge on dengue, chikungunya, and Zika diseases. It also included questions on sources of information about these diseases as well as disease transmission, disease vectors, mosquito breeding sites, experience of disease infection, management and control of diseases, and attitudes and practices toward dengue, chikungunya, and Zika prevention.

For knowledge items, a true answer was coded ‘1’, the false answer was coded ‘0’, and the total scores ranged from 0 to 4. For attitude items, a positive attitude was coded ‘1’, the negative attitude was coded ‘0’, and the total scores ranged from 0 to 9. Practice items were coded ‘1’ if the answer was ‘regular’ and ‘0’ if the answer was ‘never’. The total scores ranged from 0 to 11. Knowledge, attitude, and practice were categorized into good and poor, positive and negative attitudes, and good and bad practices, using a 50% cutoff score. A response option with a “not sure” category was provided for each question.

The third part consisted of 35 questions on knowledge of the SIT/IIT approach for vector control, such as source of information about sterile mosquitoes, how to produce sterile mosquitoes, differentiation between sterile and wild mosquitoes, benefit of application of sterile mosquitoes for vector control, frequency for release of sterile mosquitoes in the fields. For knowledge items, a true answer was coded ‘1’, a false answer was coded ‘0’, and the total scores ranged from 0 to 8. In this section, questions also included attitudes toward application of sterile mosquitoes for vector control and factors that people might take into consideration when releasing sterile mosquitoes in their households or nearby. For attitude items, a positive attitude was coded ‘1’, a negative attitude was coded ‘0’, and the total scores ranged from 0 to 6.

In addition, 18 questions on willingness to pay (WTP) were also included in this section to assess the willingness-to-pay/demand of the local residents [41] for vector control through an application of sterile mosquito release. WTP is the maximum that a person or a household would be willing to pay for a good or service [45]. To assess WTP, an artificial market was used to measure consumer preferences by directly asking their willingness to pay or willingness to accept, for exchange in the level of a good or service [46]. The total value of the good, both use and non-use values, and its flexibility facilitate valuation of a wide range of non-market goods could also be captured by this method [41]. In this study, a direct open-end format was used for uncovering value by asking respondents the maximum price that they were willing to pay for a given treatment of sterile mosquito release. The non-response rate was 65% when asking about the price that they would be willing to pay for sterile mosquitoes.

The forth part consisted of 26 questions on the impacts of a release of sterile mosquitoes on the environment, economy, society and quality of life based on the attitudes and perceptions of the respondents, such as reduction of vector control costs or insecticide applications, feeling happy and safe from disease transmission, etc.

Lastly, the fifth part included 25 questions on community engagement and acceptance of the research project such as community participation, willingness of respondents to participate in the research project, duration of the project, problems or difficulties encountered during the project, and level of acceptance and satisfaction with the project. In the last two parts, respondents were asked to score from ‘totally agree’ to ‘totally disagree’ as the highest and lowest satisfaction; ‘do not know’ was also provided for those who had no response for each specific question. The non-response rate varied in each section. For the section on knowledge about the diseases, attitudes, prevention, and practices of vector control, it ranged from 4 to 64.50%. For the section on knowledge of sterile mosquitoes and attitudes toward the application of sterile mosquitoes, it ranged from 10 to 30%. Lastly, for the section on willingness to pay, it ranged from 19.50 to 65%.

### Statistical analysis

Data was entered and cleaned using Microsoft Office Excel 2016 and statistical analysis was performed further using SPSS 18.0 (Mahidol University License (Chicago, SPSS Inc.). Descriptive summaries (frequencies and proportions) were calculated. Results were presented as Odds ratio and a 95% confidence interval (CI) was used to examine the strength of association with the main variable of interests. Chi-square tests as well as univariate logistic regressions followed by multivariate logistic regressions were performed in order to determine the factors influencing knowledge with a statistical significance of 5%; *p*-values of less or equal to 0.05 were considered significant.

## Results

### Participant demographics

In this study, 400 participants from seven communities, i.e., 180 participants from Chatuchak District (KM11: 100 participants, Khlong Prem: 40 participants, and Pahol Yothin 45: 40 participants), and 220 participants from Bang Khae District (Wat Phrom Suwan: 100 participants, Nimmannoradee: 40 participants, Yim Prayoon: 40 participants, and Perm Sap: 40 participants) were analyzed. Out of 400 participants, higher numbers of women (59.50%) read and filled out the questionnaire at home when compared to men who were usually busy working outside (Table 1). The ages of participants ranged from 18 to 87 years with a mean age of 51 years old. 43.50% of participants were married, and they were head of the family, with the highest level of education being primary school. The primary occupation of participants surveyed was government or state enterprise officer, followed by laborer, because this study was conducted in a community that largely serves the State Railway of Thailand, the state enterprise under the Ministry of Transportation. Most participants earned monthly income of 5,001–10,000 THB (US$ 142 – 285; US$ 1 = ~34 THB at the time of this study), and they lived in their own houses, followed by state enterprise residences.

**Table 1:** Demographic information of surveyed participants living in Bangkok, Thailand.

Characteristics	% (N = 400)	Median (range)
**Gender**		
Female	59.50 (238)	–
Male	40.50 (162)	–
**Age group (years)**		
18-34	13.75 (55)	27 (18–34)
35-54	35.25 (141)	46 (35-54)
> 55	38.5 (154)	62 (55-87)
Unknown/ Not answer	12.50 (50)	–
**Marital status**		
Single	24.50 (98)	–
In relationship	10.50 (42)	–
Married	43.50 (174)	–
Divorce	3.00 (12)	–
Widow/Widower	7.75 (31)	–
Unknown/ Not answer	10.75 (43)	–
**Family status**		
Head of family	38.50 (154)	–
Spouse	28.25 (113)	–
Son/daughter	9.25 (37)	–
Relatives	4.50 (18)	–
Others	4.00 (16)	–
Unknown/ Not answer	15.50 (62)	–
**Education**		
None	3.25 (13)	–
Primary school	27.50 (110)	–
Secondary school	20.50 (82)	–
High school	14.50 (58)	–
Bachelor’s degree	20.75 (83)	–
Higher than Bachelor’s degree	4.00 (16)	–
Unknown/ Not answer	9.50 (38)	–
**Occupation**		
Laborer	23.75 (95)	–
Agriculture/farmer	0.25 (1)	–
Trading/merchant	14.00 (56)	–
Government/State enterprise officer	25.00 (100)	–
Company employee	3.75 (15)	–
Housekeeper	13.50 (54)	–
Others	8.5 (34)	–
Unknown/ Not answer	11.25 (45)	–
**Monthly income (THB)** ^a^		
< 5,000	8.50 (34)	–
5,001-10,000	23.00 (92)	–
10,001-15,000	15.00 (60)	–
15,001-20,000	13.25 (53)	–
20,001-30,000	6.50 (26)	–
> 30,000	10.00 (40)	–
Unknown/ Not answer	23.75 (71)	–
**Number of family members**		
1 - 2	17.25 (69)	2 (1–2)
3 – 4	37.25 (149)	4 (3–4)
> 5	27.75 (111)	5 (5–12)
Unknown/ Not answer	17.75 (71)	–
**Characteristic of house**		
Rental house	17.25 (69)	–
Government/State enterprise residence	35.75 (143)	–
Own house	36.00 (144)	–
Unknown/ Not answer	11.00 (44)	–
**Type of house**		
House with garden	27.50 (110)	–
One-story house with high basement	9.75 (39)	–
Two-story or more house	9.75 (39)	–
Commercial building	1.50 (6)	–
Apartment/Dormitory	1.75 (7)	–
Row house	14.00 (56)	–
Others	7.00 (28)	–
Unknown/ Not answer	28.75 (115)	–

^a^1 US$ = ~34 THB

### Knowledge on dengue, chikungunya, and Zika with socio-demographic characteristics

**Fig 2:**
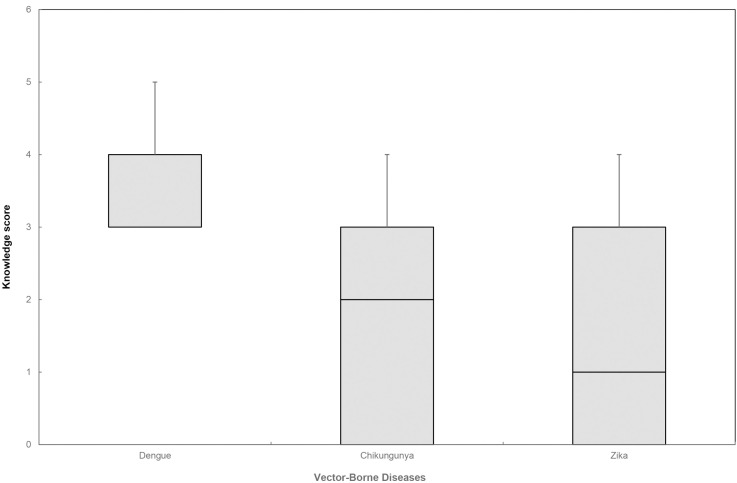
Level of knowledge on dengue, chikungunya, and Zika of participants from the questionnaire survey in Chatuchak and Bang Khae Districts, Bangkok, Thailand (4 = Excellent, 3 = Good, 2 = Moderate, 1 = Poor, and 0 = No knowledge).

Amongst the surveyed participants, 85.25% were familiar with dengue but they were less familiar with chikungunya and Zika (S1 Table, Fig 2). Participants received information about these diseases mainly from television, followed by health officials or community health volunteers. The majority of them were mostly concerned about the severity of dengue (81%); less was concerned about chikungunya and Zika. Knowledge about disease vectors, transmission and breeding sites was quite high. The vast majority of participants were able to identify *Aedes* mosquitoes as the major vector of dengue (87.25%), but much less of them were able to identify chikungunya and Zika mosquito vectors. When asked about chikungunya and Zika mosquito vectors, many participants had more doubt or they preferred not to answer, especially for Zika (S1 Table).

In this study, we could identify some significant correlations between the knowledge on dengue and socio-demographic characteristics as shown in Table 2. We found that females had significantly 1.9 times higher knowledge than males (OR = 1.905, 95%CI = 1.130-3.214, *p* = 0.016). However, we found that age, family status, marital status, education, number of family members, and monthly income had no significant correlation on dengue knowledge (*p* > 0.05). Interestingly, we found that those respondents who worked as a government or state enterprise officers had significantly 57% lower knowledge than those of laborers (OR = 0.42, 95%CI = 0.206-0.892, *p* = 0.023). Respondents who were tenants had significantly 50% lower knowledge than those of the owners of the house (OR = 0.491, 95%CI = 0.265-0.912, *p* = 0.024). Lastly, respondents with positive attitudes toward vector control, and good practices of vector control in their households, had significantly 2.9 times (OR = 2.921, 95%CI = 1.495-5.711, *p* = 0.002) and 2 times (OR = 2.027, 95%CI = 1.042-3.943, *p* = 0.037) higher knowledge on dengue than those who had negative attitudes or bad practices respectively.

**Table 2:** Analysis of the knowledge on dengue, chikungunya, and Zika with socio-demographic characteristics.

Variable	Dengue Odds Ratio (95%CI)	*P*-value	Chikungunya Odds Ratio (95%CI)	*P*-value	Zika Odds Ratio (95%CI)	*P*-value
**Gender**						
Male	Ref.		Ref.		Ref.	
Female	1.905 (1.130-3.214)	0.016^*^	1.052 (0.697-1.589)	0.808	0.821 (0.536-1.257)	0.821
**Age**						
Age < 35	Ref.		Ref.		Ref.	
Age 35–54	0.983 (0.439-2.202)	0.967	0.750 (0.394-1.427)	0.381	1.170 (0.585-2.340)	0.657
Age > 54	1.266 (0.560-2.862)	0.571	1.125 (0.601-2.106)	0.713	1.567 (0.796-3.086)	0.194
Unknown/ Not answer	0.889 (0.335-2.355)	0.813	0.706 (0.316-1.575)	0.395	0.937 (0.394-2.229)	0.883
**Family status**						
Head of family	Ref.		Ref.		Ref.	
Spouse	1.861 (0.922-3.756)	0.083	0.841 (0.512-1.382)	0.495	0.674 (0.402-1.130)	0.135
Son/daughter	1.037 (0.416-2.587)	0.938	1.043 (0.505-2.153)	0.909	0.752 (0.351-1.609)	0.463
Relative	N/A (0.000)	0.998	0.685 (0.244-1.919)	0.471	0.448 (0.141-1.424)	0.173
Others	1.048 (0.281-3.914)	0.944	1.065 (0.377-3.008)	0.905	0.940 (0.325-2.720)	0.909
Unknown/ Not answer	0.696 (0.347-1.393)	0.306	0.399 (0.203-0.785)	0.008^*^	0.376 (0.185-0.764)	0.007^*^
**Marital status**						
Single	Ref.		Ref.		Ref.	
In relationship	0.956 (0.379-2.410)	0.924	1.996 (0.960-4.150)	0.064	1.700 (0.805-3.588)	0.164
Married	1.281 (0.662-2.477)	0.462	0.983 (0.590-1.639)	0.948	1.133 (0.665-1.931)	0.645
Divorce	1.125 (0.227-5.583)	0.885	0.550 (0.140-2.160)	0.391	0.756 (0.191-2.990)	0.690
Widow	1.519 (0.472-4.883)	0.483	1.041 (0.454-2.388)	0.924	1.247 (0.532-2.922)	0.612
Unknown/ Not answer	0.655 (0.278-1.539)	0.331	0.567 (0.255-1.258)	0.163	0.518 (0.215-1.249)	0.143
**Education**						
None	Ref.		Ref.		Ref.	
Primary school	0.000 (0.000)	0.999	0.593 (0.187-1.883)	0.376	0.641 (0.201-2.041)	0.451
Secondary/ High school	0.000 (0.000)	0.999	0.662 (0.212-2.070)	0.478	0.733 (0.234-2.296)	0.593
Bachelor’s degree or higher	0.000 (0.000)	0.999	0.391 (0.121-1.259)	0.116	0.373 (0.114-1.219)	0.103
Unknown/ Not answer	0.000 (0.000)	0.999	0.194 (0.049-0.757)	0.018^*^	0.177 (0.042-0.746)	0.018^*^
**Occupation**						
Laborer	Ref.		Ref.		Ref.	
Farmer	N/A (0.000)	1.000	0.000 (0.000)	1.000	0.000 (0.000)	1.000
Merchant	1.321 (0.472-3.698)	0.596	0.907 (0.466-1.765)	0.774	1.109 (0.562-2.188)	0.765
Government/ State enterprise officer	0.429 (0.206-0.892)	0.023^*^	0.596 (0.333-1.064)	0.080	0.667 (0.365-1.219)	0.188
Company employer	0.436 (0.121-1.576)	0.206	0.605 (0.192-1.904)	0.390	0.623 (0.184-2.107)	0.447
Housekeeper	1.064 (0.397-2.854)	0.901	0.831 (0.423-1.636)	0.593	0.857 (0.424-1.731)	0.667
Others	2.537 (0.542-11.876)	0.237	0.660 (0.293-1.484)	0.315	0.714 (0.306-1.667)	0.436
Unknown/ Not answer	0.555 (0.222-1.385)	0.207	0.440 (0.203-0.954)	0.038^*^	0.555 (0.250-1.231)	0.147
**Monthly income (THB)** ^a^						
< 5,000	Ref.		Ref.		Ref.	
5,001-10,000	0.460 (0.125-1.690)	0.242	0.704 (0.319-1.551)	0.383	0.762 (0.340-1.707)	0.509
10,001-15,000	1.065 (0.238-4.759)	0.935	0.579 (0.247-1.359)	0.209	0.662 (0.276-1.585)	0.355
15,001-20,000	0.331 (0.086-1.273)	0.108	0.514 (0.213-1.240)	0.139	0.513 (0.205-1.282)	0.153
20,001-30,000	0.263 (0.061-1.140)	0.074	0.368 (0.123-1.103)	0.074	0.429 (0.137-1.340)	0.145
> 30,000	0.255 (0.065-1.007)	0.051	0.481 (0.187-1.237)	0.129	0.612 (0.234-1.601)	0.317
Unknown/ Not answer	0.516 (0.140-1.907)	0.321	0.610 (0.277-1.344)	0.220	0.692 (0.309-1.550)	0.371
**Number of family member**						
1 - 2	Ref.		Ref.		Ref.	
3 – 4	1.693 (0.812-3.531)	0.160	0.852 (0.478-1.519)	0.588	0.913 (0.506-1.649)	0.763
> 5	1.778 (0.807-3.915)	0.153	0.732 (0.396-1.353)	0.320	0.700 (0.371-1.320)	0.270
Unknown/ Not answer	0.818 (0.374-1.790)	0.615	0.584 (0.292-1.167)	0.128	0.481 (0.230-1.008)	0.053
**House ownership**						
Owner	Ref.		Ref.		Ref.	
Tenant	0.491 (0.265-0.912)	0.024^*^	1.094 (0.706-1.696)	0.688	1.006 (0.639-1.584)	0.979
Unknown/ Not answer	0.425 (0.177-1.020)	0.056	1.225 (0.614-2.443)	0.565	0.994 (0.482-2.052)	0.987
**Attitude**						
Negative	Ref.		Ref.		Ref.	
Positive	2.921 (1.495-5.711)	0.002^*^	0.594 (0.322-1.094)	0.095	0.539 (0.290-0.999)	0.050^*^
**Practice**						
Bad practice	Ref.		Ref.		Ref.	
Good practice	2.027 (1.042-3.943)	0.037^*^	1.449 (0.928-2.262)	0.103	1.360 (0.858-2.155)	0.190

* Significant different at *p* < 0.05; ^a^US$ 1 = ~34 THB

For chikungunya, we found no significant correlation between knowledge on chikungunya and gender, age, marital status, monthly income, number of family members, house ownership, attitudes, and practices (*p* > 0.05) (Table 2). However, we found that those who could not specify their family status had significantly 60% lower knowledge than those who were head of the family (OR = 0.399, 95%CI = 0.203-0.785, *p* = 0.008). Respondents who could not define or answer about their education levels had significantly 81% lower knowledge than those who were illiterate (OR = 0.194, 95%CI = 0.049-0.757, *p* = 0.018). Lastly, respondents who did not answer about their occupations had 56% lower knowledge than those who were laborers (OR = 0.440, 95%CI = 0.203-0.954, *p* = 0.038).

For Zika, we found no significant correlation between knowledge on Zika and gender, marital status, occupation, monthly income, number of family members, house ownership, and practice of vector control (*p* > 0.05) (Table 2). However, respondents who did not answer about their family status had significantly 62% lower knowledge on Zika than those who were head of the family (OR = 0.376, 95%CI = 0.185-0.764, *p* = 0.007) (Table 2). Respondents who did not answer about their educational levels had significantly 82% lower knowledge than those who had no education (OR = 0.177, 95%CI = 0.042-0.746, *p* = 0.018). Respondents with positive attitude towards vector control had significantly 46% lower knowledge on Zika than those with negative attitude (OR = 0.539, 95%CI = 0.290-0.999, *p* = 0.050).

### Prevention and control measures for dengue, chikungunya, and Zika

When asked about getting rid of breeding sites in or around their houses, the majority of participants did not answer (64.15%). However, some of them put the lids on tightly for all water containers, followed by disposing discarded water containers and garbage, and changing water in the water containers weekly (S2 Table). When asked about the methods of prevention from mosquito bites, the majority of participants did not answer (55.50%), while some chose to sleep under bed nets, followed by installing mosquito screens, using mosquito repellent coils, and turning on the fan to prevent mosquito bites.

### Attitude towards vector control with knowledge on dengue, chikungunya, and Zika

The surveyed participants almost all agreed or totally agreed to keep the house clean (91%), and to empty and scrub the water storage containers once a week (89%). However, they all agreed, or totally agreed, that elimination of mosquito breeding sites was difficult (67.50%) (S3 Table). In case of having dengue, chikungunya, and Zika patients in the house, participants believed that they had to cooperate in order to get rid of mosquito breeding sites (86.75%). Participants also believed that health officials play an important role in preventing dengue, chikungunya, and Zika infections in communities. Some of them believed that the disposal of mosquito breeding sites was the sole responsibility of health officials, while others believed the opposite. The vast majority of participants believed that sleeping under mosquito nets could prevent them from mosquito bites. They also believed that it could be life-threatening when getting infected with dengue, chikungunya, or Zika viruses without prompt treatment (84%); and the best prevention method was to prevent mosquito bites.

When we focused on knowledge and attitude, it was found that respondents with good knowledge on dengue had a significantly 2.9 times better attitude than those with poor knowledge (OR = 2.921, 95%CI = 1.495-5.711, *p* = 0.002) (Table 3). However, no significant difference in attitude was found for those with good knowledge on chikungunya (*p* > 0.05). On the contrary, those who had good knowledge on Zika had a significantly 46% worse attitude than those with poor knowledge (OR = 0.539, 95%CI = 0.322-1.094, *p* = 0.05).

**Table 3:** Analysis of knowledge on dengue, chikungunya, and Zika, and attitude toward vector control.

Variable	Good attitude % (N)	Bad attitude % (N)	Odds Ratio	95%CI	*P*-value
**Dengue**					
Poor knowledge	13.25 (53)	4.00 (16)	Ref.		
Good knowledge	75.00 (300)	7.65 (31)	2.921	1.495-5.711	0.002^*^
**Chikungunya**					
Poor knowledge	56.25 (225)	6.00 (24)	Ref.		
Good knowledge	32.00 (128)	5.75 (23)	0.594	0.322-1.094	0.095
**Zika**					
Poor knowledge	61.50 (246)	6.50 (26)	Ref.		
Good knowledge	26.75 (107)	5.25 (21)	0.539	0.290-0.99	0.050^*^

* Significant difference at *p* < 0.05

(a) good attitude refers to the scores equal to or above 4.5 out of 9 points; (b) bad attitude refers to the scores lower than 4.5 out of 9 points; (c) good knowledge refers to the scores equal to or above 2 out of 4; (d) poor knowledge refers to the scores lower than 2 out of 4

In this study, good knowledge referred to the ability to identify disease mosquito vectors, mode of disease transmission, mosquito breeding sites and their biting time. Good knowledge corresponded to scores equal to or above 2 out of 4 points, while bad knowledge corresponded to scores below 2 points. Good attitude referred to attitudes in keeping the house clean, eliminating of mosquito breeding sites, avoiding from mosquito bites, and cooperation in eliminating mosquito breeding sites. Good attitude corresponded to scores equal to or above 4.5 out of 9 points, while bad attitude corresponded to scores below 4.5 points.

### Practice of vector control measures with attitude towards vector control

The vast majority of participants always or occasionally practiced prevention and control measures at home. The majority of them had experienced having mosquito larvae in their water storage containers (S4 Table). When finding larvae, the majority of participants always or occasionally removed larvae from the containers (69.25%) or added the abate larvicide in water containers (56.25%) when finding larvae inside, while many of them did nothing. Most participants always or occasionally cleaned water containers when they found larvae (84.50%). In addition, the majority of them always or occasionally change water and wash flower vases, spotted betel vases, plant pot saucers weekly. However, less participants occasionally or always changed water or added vinegar or detergent or salt in pantry leg saucers every week to get rid of mosquito larvae. When finding mosquito breeding sites, (i.e., garbage, coconut shells, cans, and tires), the majority of participants occasionally or always turned them upside down in order to get rid of the breeding sites. They also applied guppy fishes in water containers (46.75%), while many of them did nothing. Many participants never covered water containers with lids (47.75%) while some covered them. Lastly, 68.50% of participants slept under mosquito nets, or used mosquito repellent coils while others never did.

When we focused on attitudes and practices on vector control, it was found that respondents with a good attitude on vector control significantly practiced more frequent activities on vector control than those with a bad attitude (*p* < 0.05), i.e., removal of larvae from water containers, adding the abate larvicide in water containers, changing water and washing flower vases on a weekly basis, changing water/adding vinegar/detergent in pantry leg saucers weekly, sleeping under a mosquito net at night, and using mosquito repellent coils to repel mosquitoes (Table 4). However, we found no significant difference between those with good and bad attitudes regarding the application of guppy fishes in the water containers and using the lids to cover water containers (*p* > 0.05).

**Table 4:** Analysis of attitudes and practices on vector control.

Characteristics	Good attitude % (N = 353)	Bad attitude % (N = 47)	Odds Ratio	95%CI	*P*-value
**Have you ever explored mosquito larvae in drinking water containers, cement basins in bathrooms/ toilets, or other water storage containers?**					
Never did	12.25 (49)	1.75 (7)	Ref.		
Always	34.00 (136)	5.25 (21)	0.925	0.370-2.311	0.686
Occasionally	36.25 (145)	4.50 (18)	1.151	0.454-2.920	0.768
Not eligible	1.50 (6)	0 (0)	N/A	0.000	0.999
Unknown/Not answer	4.25 (17)	0.25 (1)	2.429	0.278-21.200	0.422
**If you find larvae in drinking water containers, cement basins in bathrooms/ toilets or other water storage containers, have you ever:**					
**(1) Remove larvae**					
Never did	14.00 (56)	5.50 (22)	Ref.		
Always	39.50 (158)	1.00 (4)	15.518	5.124-46.997	0.000^*^
Occasionally	25.25 (101)	3.50 (14)	2.834	1.345-5.972	0.006^*^
Not eligible	1.00 (4)	1.00 (4)	0.393	0.090-1.710	0.213
Unknown/Not answer	8.50 (34)	0.75 (3)	4.452	1.239-16.003	0.022^*^
**(2) Add abate sand**					
Never did	27.25 (109)	4.75 (19)	Ref.		
Always	30.00 (120)	1.25 (5)	4.183	1.511-11.586	0.006^*^
Occasionally	21.75 (87)	3.25 (13)	1.167	0.546-2.494	0.691
Not eligible	1.50 (6)	1.75 (7)	0.149	0.045-0.493	0.002^*^
Unknown/Not answer	7.75 (31)	0.75 (3)	1.801	0.500-6.487	0.368
**(3) Clean water containers**					
Never did	4.75 (19)	3.25 (13)	Ref.		
Always	52.00 (208)	3.00 (12)	11.860	4.752-29.595	0.000^*^
Occasionally	26.00 (104)	3.50 (14)	5.083	2.068-12.495	0.000^*^
Not eligible	1.00 (4)	1.25 (5)	0.547	0.123-2.434	0.429
Unknown/Not answer	4.50 (18)	0.75 (3)	4.105	1.001-16.836	0.050^*^
**Do you change water and wash flower vases, spotted betel vases, plant pot saucers weekly?**					
Never did	6.50 (26)	3.50 (14)	Ref.		
Always	41.00 (164)	2.00 (8)	11.038	4.217-28.891	0.000^*^
Occasionally	32.00 (128)	3.75 (15)	4.595	1.981-10.660	0.000^*^
Not eligible	5.00 (20)	1.75 (7)	1.538	0.523-4.523	0.434
Unknown/Not answer	3.75 (15)	0.75 (3)	2.692	0.664-10.913	0.165
**Do you change water or add vinegar or detergent or salt in pantry leg saucers weekly?**					
Never did	25.50 (102)	5.75 (23)	Ref.		
Always	21.00 (84)	1.00 (4)	4.735	1.576-14.231	0.006^*^
Occasionally	34.25 (137)	3.00 (12)	2.574	1.224-5.415	0.013^*^
Not eligible	3.75 (15)	1.25 (5)	0.676	0.223-2.050	0.490
Unknown/Not answer	3.75 (15)	0.75 (3)	1.127	0.301-4.219	0.859
**Have you ever surveyed water-holding wastes such as coconut shells, cans, tires, in your household area, and have you overturned, burned, landfilled, or destroyed them weekly?**					
Never did	16.75 (67)	5.50 (22)	Ref.		
Always	28.50 (114)	1.00 (4)	9.358	3.093-28.318	0.000^*^
Occasionally	36.75 (147)	2.75 (11)	4.388	2.013-9.556	0.000^*^
Not eligible	2.50 (10)	1.50 (6)	0.547	0.178-1.679	0.292
Unknown/Not answer	3.75 (15)	1.00 (4)	1.231	0.370-4.103	0.735
**Do you put guppy fishes in water containers such as jars or cement basins?**					
Never did	38.25 (153)	5.75 (23)	Ref.		
Always	18.50 (74)	1.50 (6)	1.854	0.724-4.748	0.198
Occasionally	24.50 (98)	2.25 (9)	1.637	0.727-3.684	0.234
Not eligible	2.50 (10)	1.50 (6)	0.251	0.083-0.755	0.014^*^
Unknown/Not answer	4.50 (18)	0.75 (3)	0.902	0.246-3.305	0.876
**Do you use lids to cover all jars that are filled with water?**					
Never did	43.25 (173)	4.50 (18)	Ref.		
Always	15.25 (61)	1.25 (5)	1.269	0.452-3.566	0.651
Occasionally	20.25 (81)	3.75 (15)	0.562	0.270-1.171	0.124
Not eligible	4.75 (19)	1.50 (6)	0.329	0.117-0.931	0.036^*^
Unknown/Not answer	2.50 (10)	0.75 (3)	0.659	0.178-2.444	0.533
**Do you sleep under a mosquito net at night?**					
Never did	18.00 (72)	4.25 (17)	Ref.		
Always	40.75 (163)	1.50 (6)	6.414	2.429-16.940	0.000^*^
Occasionally	22.50 (90)	3.50 (14)	1.518	0.701-3.286	0.290
Not eligible	3.25 (13)	1.75 (7)	0.438	0.152-1.266	0.127
Unknown/Not answer	3.75 (15)	0.75 (3)	1.181	0.307-4.543	0.809
**Do you use mosquito repellent coils to repel mosquitoes?**					
Never did	12.00 (48)	4.50 (18)	Ref.		
Always	32.50 (130)	1.00 (4)	12.187	3.926-37.836	0.000^*^
Occasionally	37.75 (151)	3.50 (14)	4.045	1.872-8.738	0.000^*^
Not eligible	2.00 (8)	2.00 (8)	0.375	0.122-1.149	0.086
Unknown/Not answer	4.00 (6)	0.75 (3)	2.000	0.520-7.691	0.313

* Significant difference at *p* < 0.05

### Knowledge on sterile mosquitoes with socio-demographic characteristics

Most participants never heard about sterile mosquitoes or related issues (45.50%) but some of them had received information about it (S5 Table). When those who heard about sterile mosquitoes were asked, the major source of information was from municipal officers, followed by leaflets or flyers, community broadcast towers and local newspapers. For those who heard about sterile mosquitoes, they most likely shared it with their acquaintances. However, some of them were still uncertain to share it.

When we focused on socio-demographic characteristics, it was found that gender, age, family status, monthly income, and house ownership had no correlation with knowledge on sterile mosquitoes (*p* > 0.05) (Table 5). However, we found that marital status, education, occupation, and number of family members significantly affected knowledge (*p* < 0.05). Respondents who did not answer about their educational levels had significantly 83% lower knowledge than those without an education (OR = 0.161, 95%CI = 0.040-0.649, *p* = 0.010). Those who did not answer about their occupation had significantly 71% lower knowledge than those who were laborers (OR = 0.289, 95%CI = 0.117-0.714, *p* = 0.007). Those who did not answer about the number of their family member had significantly 65% lower knowledge than those living with 1–2 family members (OR = 0.348, 95%CI = 0.166-0.732, *p* = 0.005).

**Table 5:** Knowledge on sterile mosquitoes with socio-demographic characteristics.

Variable	Sterile mosquitoes Odds Ratio	95%CI	*P*-value
**Gender**			
Male	Ref.		
Female	0.978	0.645-1.484	0.917
**Age**			
Age < 35	Ref.		
Age 35–54	0.750	0.394-1.427	0.381
Age > 54	0.857	0.456-1.612	0.632
Unknown/Not answer	0.773	0.349-1.713	0.525
**Family status**			
Head of family	Ref.		
Spouse	0.839	0.504-1.394	0.498
Son/daughter	1.261	0.609-2.610	0.532
Relative	0.828	0.295-2.325	0.719
Others	1.287	0.455-3.643	0.634
Unknown/Not answer	0.625	0.328-1.193	0.154
**Marital status**			
Single	Ref.		
In relationship	1.043	0.502-2.166	0.911
Married	0.789	0.475-1.310	0.359
Divorce	0.695	0.196-2.464	0.573
Widow/Widower	0.334	0.126-0.887	0.028^*^
Unknown/Not answer	0.421	0.187-0.950	0.037^*^
**Education**			
None	Ref.		
Primary school	0.452	0.142-1.442	0.180
Secondary/ High school	0.447	0.142-1.405	0.168
Bachelor’s degree or higher	0.658	0.206-2.101	0.480
Unknown/Not answer	0.161	0.040-0.649	0.010^*^
**Occupation**			
Laborer	Ref.		
Farmer	2532366004	0.000	1.000
Merchant	1.014	0.516-1.995	0.967
Government/ State enterprise officer	0.738	0.409-1.329	0.311
Company employer	3.135	0.993-9.901	0.051
Housekeeper	0.784	0.389-1.579	0.495
Others	1.238	0.560-2.734	0.598
Unknown/Not answer	0.289	0.117-0.714	0.007^*^
**Monthly income (THB)** ^a^			
< 5,000	Ref.		
5,001-10,000	1.064	0.482-2.348	0.878
10,001-15,000	0.543	0.227-1.300	0.170
15,001-20,000	0.651	0.269-1.577	0.342
20,001-30,000	0.563	0.192-1.647	0.294
> 30,000	0.543	0.209-1.413	0.211
Unknown/Not answer	0.557	0.249-1.246	0.154
**Number of family member**			
1 - 2	Ref.		
3 – 4	0.829	0.464-1.478	0.524
> 5	0.704	0.381-1.303	0.264
Unknown/Not answer	0.348	0.166-0.732	0.005^*^
**House ownership**			
Owner	Ref.		
Tenant	0.902	0.580-1.402	0.646
Unknown/Not answer	0.981	0.487-1.979	0.958

* Significant difference at *p* < 0.05; ^a^1 US$ = ~34 THB

### Attitude towards the application of sterile mosquitoes and knowledge on the sterile mosquitoes

Our results showed that 47% of participants had positive attitude towards sterile mosquitoes and many of them agreed or strongly agreed that the application of sterile mosquitoes could be effective, practical and safe for human, animals, and environment. However, some of them were still uncertain on this aspect (S6 Table). Even though 32.50% of participants believed that the application of sterile mosquitoes was more practical than the use of chemicals to reduce mosquito vectors, many of them were still uncertain and some of them believed that it was less effective or even useless.

When participants were asked whether they would like to have sterile mosquitoes applied into their homes or communities, 62.75% of them desperately wanted or wanted, while some participants did not reply, some were still uncertain, and some did not required or absolutely not needed. When participants were asked to rate the overall application of sterile mosquitoes, the majority of them rated it as good to very good (52.75%), followed by moderate, did not answer, and bad to very bad. When participants were asked about their interest in implementing sterile mosquitoes in their homes or communities, the majority of them were interested or extremely interested (62.50%), followed by uncertain, did not answer, and only a few not interested or strongly not interested. When asked about factors that might influence their decision of using sterile mosquitoes in their homes or communities, most of them did not answer, followed by human safety, effectiveness, price and cost effectiveness, environmental safety, and animal safety.

When we focused on attitude and knowledge on sterile mosquitoes, it was found that there was no significant association between attitude and knowledge towards the application of sterile mosquitoes when compared between respondents with good and poor knowledge (*p* > 0.05) (Table 6). However, those with good knowledge, but still did not know whether using the sterile mosquitoes could reduce mosquitoes, had a significantly 89% worse attitude than those with poor knowledge (OR = 0.106, 95%CI = 0.040-0.282, *p* = 0.000). Those with good knowledge, and a belief that an application of sterile mosquitoes was equally useful as using chemicals, had 10 times better attitude than those with bad knowledge (OR = 10.500, 95%CI = 1.115-98.914, *p* = 0.040). Lastly, those with good knowledge, but who did not know whether they would like to have the sterile mosquitoes introduced into their households or communities, had a 68% worse attitude than those with poor knowledge (OR = 0.320, 95%CI = 0.104-0.981, *p* = 0.046).

**Table 6:** Analysis of attitude towards an application of sterile mosquitoes and knowledge on sterile mosquitoes.

Characteristics	Good knowledge %(N = 142)	Poor knowledge %(N = 258)	Odds Ratio	95%CI	*P*-value
**Do you think sterile mosquitoes are effective, practical and safe for human, animals and environment?**					
Disagree	0.50 (2)	0.50 (2)	Ref.		
Strongly agree	9.50 (38)	7.75 (31)	1.226	0.163-9.209	0.843
Agree	14.75 (59)	15.00 (60)	0.983	0.134-7.213	0.987
Not sure	7.00 (28)	22.00 (88)	0.318	0.043-2.364	0.263
Strongly disagree	0.25 (1)	0.50 (2)	0.500	0.023-11.088	0.661
Do not know/ Do not answer	3.50 (14)	18.75 (75)	0.187	0.024-1.438	0.107
**Do you think sterile mosquitoes can be used to reduce mosquito vectors of dengue, chikungunya and Zika?**					
No	3.75 (15)	13.50 (4)	Ref.		
Yes	17.00 (68)	18.75 (73)	0.869	0.391-1.934	0.732
Not sure	12.25 (49)	20.75 (83)	0.551	0.245-1.238	0.149
Do not know/ Do not answer	2.50 (10)	22.00 (88)	0.106	0.040-0.282	0.000^*^
**Do you think an application of sterile mosquitoes is more useful than chemicals in order to reduce the mosquito vectors of dengue, chikungunya and Zika?**					
Less useful	1.50 (6)	0.50 (2)	Ref.		
Useless	0.50 (2)	1.75 (7)	3.191	0.639-15.944	0.157
More useful	15.50 (62)	17.00 (68)	3.706	0.673-20.399	0.132
Equally	4.50 (18)	4.25 (17)	10.500	1.115-98.914	0.040^*^
Not sure	8.50 (34)	18.50 (74)	1.608	0.317-8.151	0.566
Do not know/ Do not answer	5.00 (20)	22.50 (90)	0.778	0.150-4.028	0.765
**If an application of sterile mosquitoes can reduce the mosquito vectors of dengue, chikungunya and Zika, would you like to have the sterile mosquitoes introduced into your household or community?**					
Not require	1.75 (7)	1.75 (7)	Ref.		
Desperately need	11.00 (44)	8.75 (35)	1.257	0.403-3.922	0.693
Need	10.00 (40)	15.50 (62)	0.645	0.210-1.978	0.443
Not sure	4.50 (18)	13.25 (53)	0.340	0.105-1.101	0.072
Absolutely not need	0.25 (1)	0.25 (1)	1.000	0.052-19.360	1.000
Do not know/ Do not answer	8.00 (32)	25.00 (100)	0.320	0.104-0.981	0.046^*^
**Could you please give rating on the application of sterile mosquitoes in order to reduce the mosquito vectors of dengue, chikungunya and Zika?**					
Absolutely not good	0.50 (2)	0.75 (3)	Ref.		
Not good	1.25 (5)	5.75 (23)	0.326	0.043-3.492	0.280
Moderate	9.00 (36)	20.75 (83)	0.651	0.104-4.062	0.646
Good	9.50 (38)	19.50 (78)	0.731	0.117-4.559	0.737
Very good	13.50 (54)	10.25 (41)	1.976	0.315-12.373	0.467
Do not know/ Do not answer	1.75 (7)	7.50 (30)	0.350	0.049-2.508	0.296
**Are you interested in implementing new technologies or methods to reduce the mosquito vectors of dengue, chikungunya and Zika in your household or community?**					
Not interested	0.50 (2)	0.75 (3)	Ref.		
Extremely interested	12.75 (51)	11.75 (47)	1.628	0.260-10.173	0.602
Interested	12.00 (48)	26.25 (105)	0.686	0.111-4.238	0.685
Not sure	7.25 (29)	13.75 (55)	0.791	0.125-5.004	0.803
Strongly not interested	0 (0)	0.50 (2)	0.000	0.000	0.999
Do not know/ Do not answer	3.00 (12)	11.50 (46)	0.391	0.059-2.613	0.333

* Significant difference at *p* < 0.05

### Willingness to pay for the application of sterile mosquitoes in order to control dengue, chikungunya, and Zika

When participants were asked to select one choice of sterile mosquito release when it was at their own expenses, the majority of them did not select any options, some of them preferred (2) sterile mosquitoes with two-week release frequency, followed by (1) sterile mosquitoes with one-week release frequency (S7 Table). For those participants who selected (1) sterile mosquitoes with one-week release frequency, most of them were not willing to purchase any sterile mosquitoes when they were available on the market. Only a few of them were willing to pay from 2.50 - 15.00 THB (~US$ 0.07 - 0.44), and the maximum that they could pay was 1 THB per one sterile mosquito. When asked why they did not want to purchase sterile mosquitoes, most of them expected to get these sterile mosquitoes as public support from the government, followed by reasons of affordability, or they needed other measures to prevent and control dengue, chikungunya and Zika.

For those who selected (2) sterile mosquitoes with two-week release frequency, most of them also were not willing to purchase any sterile mosquitoes when they were available on the market. Only a few of them were willing to pay from 2.50 – 15.00 THB (~US$ 0.07 - 0.44), and the maximum that they could pay was 2 THB (~US$ 0.06) per one sterile mosquito, which was almost double the price of those who chose (1) sterile mosquitoes with one-week release frequency (S7 Table). When asked about the reasons why they did not want to purchase sterile mosquitoes, most of them also wanted to get these sterile mosquitoes as public support from the government, which was at a much higher percentage than those who selected (1) sterile mosquitoes with one-week release frequency, followed by reasons of affordability, they wanted to know more information or scientific evidence, or they needed other measures to prevent and control dengue, chikungunya and Zika. For comparison, for those who selected (2) sterile mosquitoes with two-week release frequency, they wanted to know more information in a much higher percentage than those who selected sterile one-week release frequency.

## Discussion

This study revealed that participants living in Bangkok were more familiar with dengue (85.25%) and at a much higher level than those familiar with chikungunya (39.75%) or Zika (33.75%). Level of knowledge on dengue was the highest amongst the studied diseases. This finding is supported by Saminpanya and Jarujit who reported high levels of knowledge among respondents towards dengue (71%) when compared to those of chikungunya (23%) [47]. Dengue has been endemic for more than 60 years in Thailand [48], and it has been recognized as a major public health problem [49] since the first major outbreak of dengue in Thailand in 1958 [50,51]. Moreover, dengue virus infections usually occur with common severe presentation of hemorrhagic manifestations and occasional deaths from shock [51], thus it has remained a significant threat to the welfare of the Thai population [52]. Although a chikungunya outbreak was also reported in 1958 [53,54], little was known, so the disease received little attention as it had generally been regarded as self-limiting with few severe complications [55].

The first report of the possible occurrence of Zika virus in Thailand was published in 1963 [56] and Zika virus has been circulating in Thailand since 2012 [57]. However, Zika virus infections represent a small proportion of ongoing flaviviral infections in Thailand [52], with the exception of rare cases of more severe disease [58]. In addition, Zika was a relatively mild and self-limiting disease, which was often resolved without medical attention [59], thus little was known for Zika, especially to the general public.

In this study, participants had a generally high knowledge of mosquito vectors and vector-borne diseases, i.e., they could identify at least one mosquito breeding site, they could correctly identify biting time of *Aedes* mosquitoes, and they considered dengue, chikungunya, and Zika as severe diseases, even though they did not experience virus infections themselves. When we focused on socio-demographics, we found that females had a higher knowledge on dengue than males, but this was not the case for chikungunya and Zika. Age, education, family status, marital status, monthly income, and number of family members were not associated with the level of knowledge on dengue, chikungunya and Zika. Our study was supported by Elson et al. who found a greater knowledge of dengue in females when compared to males in Peru [60]. So et al. supported that there was no correlation between education, age, marital status, family income and knowledge levels [61]. However, our findings together with So et al. contradicted other studies that showed good correlation between knowledge and socio-demographic information, i.e., age, education, marital status, employment and monthly income [62–65]. In this study, we found that most people had good knowledge on dengue, and this might be the reason why we found no association between knowledge on dengue and socio-demographic characteristics.

In terms of prevention and control measures for vector control, covering water storage containers was the most common preventive measure cited by participants, followed by disposal of mosquito breeding sites such as discarded water containers or garbage, and weekly change of water in water storage containers. These results are supported by the study of Swaddiwudhipong et al. [66]. High number of *Aedes* mosquitoes was present in water storage containers, and large water storage containers represented 80% of *Aedes* breeding sites [67]. This could explain why participants might be aware of treating these water storage containers [68].

When we focused on attitude, although the majority of participants had good attitude in many aspects, such as regularly changing water in water storage containers, sleeping under a bed net, and preventing themselves from mosquito bites, many still believed that elimination of mosquito breeding sites was the sole responsibility of the health officials. Similarly, Kamaran et al. showed that people were less likely to apply vector control measures despite its benefits since the government had performed vector control measures for a long time and people believed that it was the government’s responsibility [68].

Other studies observed good knowledge and poor practice [60,62,68]. Although we found that good knowledge on dengue was positively associated with good attitude, and good attitude was associated with good practice, when we focused on a specific activity, such as covering water storage containers to get rid of mosquito breeding sites, many participants had never practiced it (47.75%). The reason why participants rarely covered water storage containers might be the fact that they lived in urban or suburban areas in Bangkok where they could easily access a water supply without storing water, hence, less water storage containers are found. However, we generally found that participants with a good attitude practiced more frequent activities in vector control. These findings are supported by Al-Dubai et al., who found an association between knowledge and practice in dengue control [69].

High level of knowledge has been related to the diffusion of information [33]; and the media has played a major role in the dissemination of public health knowledge [32,70,71]. In this study, we found that television was one of the major sources of information on dengue, chikungunya and Zika, followed by health officers or community health volunteers. Our findings are supported by Al-Dubai et al., who also showed that TV was the main source of information on vector-borne diseases in Malaysia [69]; and Swaddiwudhipong et al. who revealed the important role of health personnel in disseminating information and prevention methods of dengue in Thailand [66]. Village health volunteers or community health volunteers have played an important role in facilitating effective health activities that increase awareness, motivation, involvement and monitoring health status within a community [72–74]; and together with primary health care units, they have been the public’s first point of contact with primary health care and the broader health system in Thailand [48].

Regarding knowledge on sterile mosquitoes and relevant information, many participants knew nothing about sterile mosquitoes while a few had some knowledge, such as how to sterilize mosquitoes, benefits of sterile mosquitoes, difference between sterile and wild mosquitoes, etc. Our study highlighted the importance of communication channels through municipal officials and community health volunteers in Bangkok, Thailand. Municipal officials were the major source of information about sterile mosquitoes, followed by leaflets or flyers, and community broadcast towers. More efforts have to be done in order to deliver more information and knowledge on sterile mosquitoes through the previously mentioned channels of communication in order to reach more people in the community prior to the release or implementation of sterile mosquitoes in the fields. Moreover, various media channels, i.e., TV and online media, are needed in order to reach more people and the general public. Good communication strategies to inform target communities in order to solicit their acceptance and support are essential for successful implementation of a mosquito control program that includes an SIT component [28,75,76].

When we focused on knowledge of sterile mosquitoes and socio-demographic characteristics, we found no association amongst them. These findings could be explained by the fact that the majority of participants had poor knowledge on sterile mosquitoes; and since this technology is still new in Thailand (together with limited or less channels of communication to deliver key messages on the technology), no correlation was found in this study. Also, when we focused on attitudes toward sterile mosquitoes or the application of sterile mosquitoes to reduce mosquito vectors of dengue, chikungunya and Zika, we found no association between attitude and level of knowledge on sterile mosquitoes. A poor knowledge on sterile mosquitoes, as previously described, could be explained by the same reason. Although many participants showed a positive attitude towards sterile mosquitoes, i.e., they were interested in having sterile mosquitoes released in their homes or communities, and they believed that it could reduce chemical use and reduce the *Aedes* mosquito populations in their homes or communities, a non-negligible percentage of participants was still uncertain about sterile mosquitoes. In comparison, the majority of respondents from the questionnaire survey in Greece agreed that SIT has many advantages over chemical control methods; however, their attitudes toward SIT still ranged from indecisive to fully supportive [28].

In this study, there were some limitations attributed to the methodology and the questionnaire surveys. As all information was acquired via self-reporting questionnaire (S1 File), respondents might have provided answers that were not reflective of their actual attitudes and practices but to satisfy social expectations, which might contribute to reporting bias. In addition, we had limitations in the result interpretation in some sections because of a high non-response rate of more than 50%. To improve this study, face-to-face interviews by trained interviewers in order to prove and clarify the responses of respondents should be conducted, which in turn could help increase knowledge and basic information on SIT/IIT technology. Basically, we first aimed to conduct face-to-face interviews, but it was not possible since this study was done during the period when the COVID-19 pandemic occurred and access to the communities was restricted or almost impossible. In Greece, the presence of key persons, i.e., scientific experts and municipality members, was shown to raise community awareness and might prove important in granting permission to enter their private properties for entomological surveillance [28]. Another limitation in our study was that the periods when respondents answered the questionnaire were different in each community, and it took a year to complete, while it should take 1–3 months for face-to-face interviews. This might have affected the opinions or perceptions of respondents. In addition, since this study used a non-probability sampling method for data collection, it might introduce potential selection bias that could lead to over or under representation of certain groups of participants; as a result, our findings might not be generalized to a broader community.

Besides, the level of education might be another limitation of this study since some questions required a higher level of education to be comprehensive and responsive. Although most respondents in this study were educated to the secondary or high school level, others received an education only through primary school. Higher education leads to better and easier access to information, and has an indirect effect on knowledge, reflected by the ability to understand and comprehend complex information [77]. In this study, we found that answers ranged from non-negligible to high percentages of non-response in each section of the questionnaires, and this could be due to the fact that respondents had low education or had difficulty understanding and answering the questionnaire. Less knowledge on chikungunya, Zika, sterile mosquitoes and poor practices on vector control were also observed in those who showed no response in this study. Moreover, as this study was carried out only in Chatuchak and Bang Khae Districts, and most respondents had their education in the primary or secondary schools, the results might only represent information about these studied groups of people, and it might not represent those with higher education or those living in other parts of Bangkok. Lastly, before the questionnaire survey, we conducted community engagement and knowledge about sterile mosquitoes was partly delivered through community health volunteers. Therefore, people in the studied communities might have higher or more knowledge about sterile mosquitoes than the general public.

Overall, this study was the first survey examining community knowledge and acceptance of the SIT/IIT technology alongside traditional control methods in Thailand. Results from this study showed that most people in Bangkok were more familiar and had better knowledge on dengue than those of chikungunya and Zika. However, there was no association between socio-demographic factors and knowledge on dengue, chikungunya, and Zika. A strong association between good knowledge on dengue, good attitude, and good practice were observed, but this was not the case for chikungunya and Zika. Generally, the majority of people had poor knowledge on sterile mosquitoes, and no association between socio-demographic factors and knowledge on sterile mosquitoes was found. Also, there was no association between knowledge and attitude on sterile mosquitoes. More information on vector-borne diseases, mosquito vectors, and their prevention and control should be provided to the general public by health authorities in order to increase the level of knowledge. In addition, more efforts in communication and community engagement are required in order to educate people about sterile mosquitoes and SIT/IIT technology before implementing it at a larger scale in Bangkok.

Our study was the first willingness to pay (WTP) analysis of SIT/IIT deployment for mosquito control. Prior to this, WTP analysis was conducted only for fruit fly control using the SIT approach. Moreover, this study provides unique insights into about how local residents in the capital city of Bangkok viewed SIT/IIT technology. Although many residents still lack of knowledge on the technology, they were willing to accept or would like to have this new technology implemented in their communities. This was because they considered that the available conventional mosquito control methods were not effective enough and this technology could be an alternative mosquito control method. Many residents were willing to receive the sterile mosquitoes if they were public supported by government authorities. However, many of them were less likely to purchase the sterile mosquitoes if the costs were on their own expenses. For those few of them who were willing to pay, only 9.0-9.5% of them could afford to purchase sterile mosquitoes at a price of 1–2 THB (~US$ 0.03-006) per sterile mosquito. These findings highlighted the funding challenges for SIT/IIT mosquito control programs, as it emphasized the need for government subsidization of the costs and the need to explore alternative funding strategies to ensure the program sustainability, which is a very big challenge for developing countries. Collaboration or continued support from the government is vital for strengthening a successful long-term SIT/IIT large-scale vector control program; as was the case in Singapore [78].

## Supporting information

S1 Table Knowledge on dengue, chikungunya and Zika of the surveyed participants living in Bangkok, Thailand. (PDF)

S2 Table Prevention and control measures for dengue, chikungunya, and Zika of the surveyed participants living in Bangkok, Thailand. (PDF)

S3 Table Attitudes toward dengue, chikungunya, and Zika of the surveyed participants living in Bangkok, Thailand. (PDF)

S4 Table Practices in vector control by the surveyed participants living in Bangkok, Thailand. (PDF)

S5 Table Knowledge on sterile mosquitoes of the surveyed participants living in Bangkok, Thailand. (PDF)

S6 Table Attitudes toward the application of sterile mosquitoes of surveyed participants living in Bangkok, Thailand. (PDF)

S7 Table Willingness to pay (WTP) for the application of sterile mosquitoes to reduce mosquito vectors of dengue, chikungunya, and Zika of the surveyed participants living in Bangkok, Thailand. (PDF)

S1 File Questionnaire form that is translated in English. (DOCX)

S1 Data Data used to generate S1 - S7 Tables. (XLSX)
