# Supplementary material for: Knowledge, attitude, and practice (KAP), and acceptance and willingness to pay (WTP) for mosquito-borne diseases control through sterile mosquito release in Bangkok, Thailand
Source: PLoS Negl Trop Dis. 2025 Jul 28;19(7):e0011935. doi: 10.1371/journal.pntd.0011935 (PMC12303319; doi:10.1371/journal.pntd.0011935)
Supplement: S1 Table — (PDF) [file pntd.0011935.s001.pdf]

S1 Table. Knowledge on dengue, chikungunya and Zika of the surveyed participants living in Bangkok, Thailand.

**Characteristics**			
	**Dengue**	**Chikungunya**	**Zika**
**Have you heard about these diseases?**	**% (N = 400)**	**% (N = 400)**	**% (N = 400)**
Yes	85.25 (341)	39.75 (159)	33.75 (135)
Never	6.50 (26)	35.00 (140)	39.75 (159)
Unknown/Not answer	8.25 (33)	25.25 (101)	26.50 (106)
**What are your sources of information about these diseases? (Multiple answers)**			
Radio	11.25 (45)	5.25 (21)	4.00 (16)
Television	45.00 (180)	23.75 (95)	20.25 (81)
Newspapers	3.25 (13)	0.75 (3)	0.75 (3)
Online Media	9.25 (37)	6.50 (26)	6.75 (27)
Poster / Brochure	0.75 (3)	0 (0)	0.25 (1)
Health official/Village health volunteer	20.75 (83)	19.00 (76)	18.00 (72)
Neighbor / Acquaintance	2.00 (8)	2.50 (10)	1.25 (5)
Workplace / School	1.50 (6)	0.50 (2)	0.25 (1)
Unknown / Not answer	6.25 (25)	41.75 (167)	48.50 (194)
**Do you think these diseases are severe?**			
Severe	81.00 (324)	42.50 (170)	37.00 (148)
Mild	4.50 (18)	8.25 (33)	6.25 (25)
Unknown/Not answer	14.50 (58)	49.25 (197)	56.75 (227)
**What kinds of mosquitoes carry these diseases?**			
Aedes mosquitoes	87.25 (349)	42.25 (169)	35.50 (142)
Culex mosquitoes	0.75 (3)	0.50 (2)	1.25 (5)
Anopheles mosquitoes	0.50 (2)	4.75 (19)	1.00 (4)
All kinds of mosquitoes	4.25 (17)	7.00 (28)	10.25 (41)
Unknown/Not answer	7.25 (29)	45.50 (182)	52.00 (208)
**Identify breeding sites of *Aedes* mosquitoes**			
Stagnant water	78.50 (314)	43.25 (173)	39.25 (157)
Flowing tides	1.50 (6)	1.50 (6)	0.73 (3)
Dirty places	2.25 (9)	3.50 (14)	3.50 (14)
Water containers	11.25 (45)	6.00 (24)	5.25 (21)
Wasteland	0.25 (1)	1.25 (5)	1.00 (4)
Garbage disposal areas	0.50 (2)	0.75 (3)	0.50 (2)
Drainage pipe system	1.50 (6)	2.00 (8)	2.00 (8)
Unknown/Not answer	4.25 (17)	41.75 (167)	47.75 (191)
**How are these diseases transmitted from person to person?**			
By mosquito bites	81.00 (324)	46.75 (187)	40.50 (162)
By touching each other	3.00 (12)	2.25 (9)	2.25 (9)
By water	2.00 (8)	1.50 (6)	1.00 (4)
By flies	0.50 (2)	0.50 (2)	0.75 (3)
By other animals	2.00 (8)	1.25 (5)	2.00 (8)
Unknown/Not answer	11.50 (46)	47.75 (191)	53.50 (214)
**When do you think *Aedes* mosquitoes bite people?**			
Day	64.00 (265)	36.00 (144)	31.00 (124)
Night	5.25 (21)	4.00 (16)	3.50 (14)
Both day and night	20.00 (80)	13.00 (52)	11.50 (46)
Evening / Dusk	3.75 (15)	3.00 (12)	2.75 (11)
Unknown / Not answer	7.00 (28)	44.00 (176)	51.25 (205)
**Have you ever suffered from these diseases?**			
Yes	18.75 (75)	2.25 (9)	1.00 (4)
No	71.00 (284)	64.75 (259)	63.00 (252)
Unknown/Not answer	10.25 (41)	33.00 (132)	36.00 (144)
**Do you have any acquaintances who have suffered from these diseases?**			
Yes	48.25 (193)	14.00 (56)	6.00 (24)

Characteristics			
	**Dengue**	**Chikungunya**	**Zika**
No	41.00 (164)	47.75 (191)	51.50 (206)
Unknown / Not answer	10.75 (43)	38.25 (153)	42.50 (170)
