# Supplementary material for: Knowledge, attitude, and practice (KAP), and acceptance and willingness to pay (WTP) for mosquito-borne diseases control through sterile mosquito release in Bangkok, Thailand
Source: PLoS Negl Trop Dis. 2025 Jul 28;19(7):e0011935. doi: 10.1371/journal.pntd.0011935 (PMC12303319; doi:10.1371/journal.pntd.0011935)
Supplement: S2 Table — (PDF) [file pntd.0011935.s002.pdf]

S2 Table. Prevention and control measures for dengue, chikungunya, and Zika of the surveyed participants living in Bangkok, Thailand.

**Characteristics**	**% (N = 400)**
**How do you get rid of the breeding sites for mosquito larvae in and around your homes? (Multiple answers)**	
Put lids on all water containers tightly	11.25 (45)
Change water in the water containers weekly	5.85 (23)
Discarded water container disposal/ garbage disposal	8.65 (35)
Brush and scrub inside the water containers	1.85 (7)
Release fish to consume larvae in water containers	2.75 (11)
Put larvicides/chemicals in water containers	2.75 (11)
Scoop mosquito larvae out of water containers	1.10 (4)
Did not do anything	1.10 (4)
Unknown	0.65 (3)
Not answer	64.15 (257)
**What methods do you use to protect yourself and your family members from mosquito bites? (Multiple answers)**	
Sleep under mosquito nets	9.50 (38)
Use mosquito repellent coils	7.55 (30)
Install mosquito screens	8.55 (34)
Turn on the fan to prevent mosquito bites	7.35 (29)
Use a mosquito shock machine/ mosquito mat	3.75 (15)
Use insecticide-impregnated mosquito nets	0.70 (3)
Apply mosquito repellent lotion when entering the forest/ garden	4.25 (17)
Wear long-sleeved shirts and long pants when entering the forest/garden	1.50 (6)
Did not do anything	0.25 (1)
Others	0.35 (1)
Unknown	0.75 (3)
Not answer	55.50 (222)
