# Supplementary material for: Knowledge, attitude, and practice (KAP), and acceptance and willingness to pay (WTP) for mosquito-borne diseases control through sterile mosquito release in Bangkok, Thailand
Source: PLoS Negl Trop Dis. 2025 Jul 28;19(7):e0011935. doi: 10.1371/journal.pntd.0011935 (PMC12303319; doi:10.1371/journal.pntd.0011935)
Supplement: S3 Table — (PDF) [file pntd.0011935.s003.pdf]

S3 Table. Attitudes toward dengue, chikungunya, and Zika of the surveyed participants living in Bangkok, Thailand.

Characteristics	% (N = 400)
**It is essential to keep the house and surrounding areas clean.**	
Totally agree	61.75 (247)
Agree	29.25 (117)
Uncertain	4.50 (18)
Disagree	0 (0)
Strongly disagree	0 (0)
Unknown/Not answer	4.50 (18)
**It is the right thing to do to empty and scrub water storage containers once a week.**	
Totally agree	53.00 (121)
Agree	36.00 (144)
Uncertain	6.75 (27)
Disagree	0 (0)
Strongly disagree	0 (0)
Unknown/Not answer	4.25 (17)
**It is difficult to eliminate breeding sites of the mosquito vectors of dengue, chikungunya and Zika viruses.**	
Totally agree	36.25 (145)
Agree	31.25 (125)
Uncertain	18.25 (73)
Disagree	7.75 (31)
Strongly disagree	1.25 (5)
Unknown/Not answer	5.25 (21)
**Households with dengue, chikungunya and Zika patients must cooperate to eliminate mosquito breeding sites**	
Totally agree	45.25 (181)
Agree	41.50 (166)
Uncertain	7.25 (29)
Disagree	1.25 (5)
Strongly disagree	0.25 (1)
Unknown/Not answer	4.50 (18)
**Health officials play a critical role in preventing dengue, chikungunya, and Zika at the community level**	
Totally agree	46.50 (186)
Agree	35.75 (143)
Uncertain	9.50 (38)
Disagree	3.25 (13)
Strongly disagree	0.50 (2)
Unknown/Not answer	4.50 (18)
**Disposal of mosquito breeding sites is the sole responsibility of health officials**	
Totally agree	20.75 (83)
Agree	15.00 (60)
Uncertain	19.25 (77)
Disagree	28.75 (115)
Strongly disagree	11.25 (45)
Unknown/Not answer	5.00 (20)
**Sleeping under mosquito nets can prevent dengue, chikungunya and Zika viruses**	
Totally agree	26.50 (106)
Agree	37.25 (149)
Uncertain	25.50 (102)
Disagree	5.25 (21)
Strongly disagree	1.00 (4)
Unknown/Not answer	4.50 (18)
**It could be life-threatening if you are sick with dengue or chikungunya or Zika and do not**	

Characteristics	% (N = 400)
**treated quickly**	
Totally agree	45.25 (181)
Agree	38.75 (155)
Uncertain	10.50 (42)
Disagree	0.50 (2)
Strongly disagree	0.75 (3)
Unknown/Not answer	4.25 (17)
**The best method to prevent dengue, chikungunya and Zika is to avoid mosquito bites**	
Totally agree	42.00 (168)
Agree	36.50 (146)
Uncertain	15.25 (61)
Disagree	1.00 (4)
Strongly disagree	0 (0)
Unknown/Not answer	5.25 (21)
