# Supplementary material for: Knowledge, attitude, and practice (KAP), and acceptance and willingness to pay (WTP) for mosquito-borne diseases control through sterile mosquito release in Bangkok, Thailand
Source: PLoS Negl Trop Dis. 2025 Jul 28;19(7):e0011935. doi: 10.1371/journal.pntd.0011935 (PMC12303319; doi:10.1371/journal.pntd.0011935)
Supplement: S4 Table — (PDF) [file pntd.0011935.s004.pdf]

S4 Table. Practices in vector control by the surveyed participants living in Bangkok, Thailand.

Characteristics	% (N = 400)
**Have you ever explored mosquito larvae in drinking water containers, cement basins in bathrooms / toilets, or other water storage containers?**	
Always	39.25 (157)
Occasionally	40.75 (163)
Never did	14.00 (56)
Not eligible	1.50 (6)
Unknown/Not answer	4.50 (18)
**If you find larvae in drinking water containers, cement basins in bathrooms / toilets or other water storage containers, have you ever:**	
**(1) Remove larvae**	
Always	40.50 (162)
Occasionally	28.75 (115)
Never did	19.50 (78)
Not eligible	2.00 (8)
Unknown/Not answer	9.25 (37)
**(2) Add abate sand**	
Always	31.25 (125)
Occasionally	25.00 (100)
Never did	32.00 (128)
Not eligible	3.25 (13)
Unknown/Not answer	8.50 (34)
**(3) Clean water containers**	
Always	55.00 (220)
Occasionally	29.50 (118)
Never did	8.00 (32)
Not eligible	2.25 (9)
Unknown/Not answer	5.25 (21)
**Do you change water and wash flower vases, spotted betel vases, plant pot saucers weekly?**	
Always	43.00 (172)
Occasionally	35.75 (143)
Never did	10.00 (40)
Not eligible	6.75 (27)
Unknown/Not answer	4.50 (18)
**Do you change water or add vinegar or detergent or salt in pantry leg saucers weekly?**	
Always	22.00 (88)
Occasionally	37.25 (149)
Never did	31.25 (125)
Not eligible	5.00 (20)
Unknown/Not answer	4.50 (18)
**Have you ever surveyed water-holding wastes such as coconut shells, cans, tires, in your household area, and have you overturned, burned, landfilled, or destroyed them weekly?**	
Always	29.50 (118)
Occasionally	39.50 (158)
Never did	22.25 (89)
Not eligible	4.00 (16)
Unknown/Not answer	4.75 (19)
**Do you put guppy fishes in water containers such as jars or cement basins?**	
Always	20.00 (80)
Occasionally	26.75 (107)
Never did	44.00 (176)
Not eligible	4.00 (16)
Unknown/Not answer	5.25 (21)
**Do you use lids to cover all jars that are filled with water?**	

Characteristics	% (N = 400)
Always	16.50 (66)
Occasionally	24.00 (96)
Never did	47.75 (191)
Not eligible	6.25 (25)
Unknown/Not answer	5.50 (22)
**Do you sleep under a mosquito net at night?**	
Always	42.25 (169)
Occasionally	26.00 (104)
Never did	22.25 (89)
Not eligible	5.00 (20)
Unknown/Not answer	4.50 (18)
**Do you use mosquito repellent coils to repel mosquitoes?**	
Always	42.25 (169)
Occasionally	26.00 (104)
Never did	22.25 (89)
Not eligible	5.00 (20)
Unknown/Not answer	4.50 (18)
