# Supplementary material for: Knowledge, attitude, and practice (KAP), and acceptance and willingness to pay (WTP) for mosquito-borne diseases control through sterile mosquito release in Bangkok, Thailand
Source: PLoS Negl Trop Dis. 2025 Jul 28;19(7):e0011935. doi: 10.1371/journal.pntd.0011935 (PMC12303319; doi:10.1371/journal.pntd.0011935)
Supplement: S5 Table — (PDF) [file pntd.0011935.s005.pdf]

S5 Table. Knowledge on sterile mosquitoes of the surveyed participants living in Bangkok, Thailand.

Characteristics	% (N = 400)
**Have you heard about sterilization of mosquitoes to reduce the mosquito vectors of dengue, chikungunya and Zika?**	
Yes	37.75 (151)
No	45.50 (182)
Unknown/Not answer	16.75 (67)
**If yes, where did you receive information about sterilization of mosquitoes in order to reduce the mosquito vectors of dengue, chikungunya, and Zika? (Multiple answers)**	
Local newspapers	12.57 (24)
Village broadcast tower	13.61 (26)
Municipal Officer	19.90 (38)
Billboard	12.04 (23)
Leaflet / Flyer	16.75 (32)
Others	10.47 (20)
Unknown	14.66 (28)
**Do you think you would provide information about mosquito sterilization to your family, acquaintances and neighbors?**	
Yes	58.50 (234)
No	4.75 (19)
Not sure	14.50 (58)
Unknown	11.75 (47)
Not answer	10.50 (42)
**What kinds of mosquitoes are used for sterilization?**	
All kinds of mosquitoes	24.75 (99)
Aedes mosquitoes	32.50 (130)
Culex mosquitoes	0.25 (1)
Anopheles mosquitoes	1.25 (5)
Others	0 (0)
Unknown	35.00 (140)
Not answer	6.25 (25)
**Which of the followings are the methods used to sterilize *Aedes* mosquitoes?**	
Low dose irradiation	7.50 (30)
Injection of bacteria that resist dengue/ chikungunya/ Zika viruses into mosquitoes	7.00 (28)
Both are correct	29.50 (118)
Unknown	46.50 (186)
Not answer	9.50 (38)
**What sex of *Aedes* mosquitoes is used for sterilization?**	
Males	24.25 (97)
Females	14.75 (59)
Both males and females	19.50 (78)
Unknown	36.50 (146)
Not answer	5.00 (20)
**What is the difference between *Aedes* males and females?**	
Males do not feed on blood, only on nectar	28.25 (113)
Females feed on blood, carrying dengue fever	33.25 (133)
Males have thick and long antennae	8.75 (35)
Females do not have thick and long antennae	0.25 (1)
All of the above	4.50 (18)
More than one item	1.75 (7)
Not sure	5.75 (23)
Unknown	13.75 (55)
Not answer	3.75 (15)

Characteristics	% (N = 400)
**How sterile mosquitoes are different from wild mosquitoes?**	
1) Sterile mosquitoes are able to resist germs	11.25 (45)
2) Sterile mosquitoes are unable to mate with wild mosquitoes	6.25 (25)
3) Sterile mosquitoes can mate with wild mosquitoes but cannot produce offspring	11.75 (47)
4) Both 1 and 3 are correct	14.25 (57)
5) All are correct	10.25 (41)
Unknown	36.25 (145)
Not answer	10.00 (40)
**Which of the followings are correct?**	
1) Sterile mosquitoes can produce offspring	6.00 (24)
2) Sterile mosquitoes are only female	3.75 (15)
3) Sterile mosquitoes are not able to produce offspring	12.50 (50)
4) Sterile mosquitoes are only male	5.25 (21)
5) Both 3 and 4 are correct	21.00 (84)
All are wrong	0.75 (3)
Unknown	40.00 (160)
Not answer	10.75 (43)
**What are the benefits of sterile mosquitoes?**	
1) Help reducing mosquito populations in household / community	14.00 (56)
2) Reduce the risk of dengue / chikungunya / Zika	9.25 (37)
3) Both 1 and 2 are correct	32.75 (131)
All are wrong	1.75 (7)
Unknown	30.75 (123)
Not answer	11.50 (46)
**How often to release sterile mosquitoes in order to reduce mosquito populations in nature?**	
Every week	21.25 (85)
Every two weeks	14.50 (58)
Every month	9.50 (38)
Not sure	17.25 (69)
Unknown	27.50 (110)
Not answer	10.00 (40)
