# Supplementary material for: Knowledge, attitude, and practice (KAP), and acceptance and willingness to pay (WTP) for mosquito-borne diseases control through sterile mosquito release in Bangkok, Thailand
Source: PLoS Negl Trop Dis. 2025 Jul 28;19(7):e0011935. doi: 10.1371/journal.pntd.0011935 (PMC12303319; doi:10.1371/journal.pntd.0011935)
Supplement: S6 Table — (PDF) [file pntd.0011935.s006.pdf]

S6 Table. Attitudes toward the application of sterile mosquitoes of surveyed participants living in Bangkok, Thailand.

Characteristics	% (N = 400)
**Do you think sterile mosquitoes are effective, practical and safe for human, animals and environment?**	
Strongly agree	17.25 (69)
Agree	29.75 (119)
Not sure	29.00 (116)
Disagree	1.00 (4)
Strongly disagree	0.75 (3)
Do not know / Do not answer	22.25 (89)
**Do you think sterile mosquitoes can be used to reduce mosquito vectors of dengue, chikungunya and Zika?**	
Yes	35.25 (141)
No	7.25 (29)
Not sure	33.00 (132)
Do not know/ Do not answer	24.50 (98)
**Do you think an application of sterile mosquitoes is more useful than chemicals in order to reduce the mosquito vectors of dengue, chikungunya and Zika?**	
More useful	32.50 (130)
Equally	8.75 (35)
Less useful	2.00 (8)
Useless	2.25 (9)
Not sure	27.00 (108)
Do not know/ Do not answer	27.50 (110)
**If an application of sterile mosquitoes can reduce the mosquito vectors of dengue, chikungunya and Zika, would you like to have the sterile mosquitoes introduced into your household or community?**	
Desperately need	19.75 (79)
Need	25.50 (102)
Not sure	17.75 (71)
Not require	3.50 (14)
Absolutely not need	0.50 (2)
Do not know/ Do not answer	33.00 (132)
**Could you please give rating on the application of sterile mosquitoes in order to reduce the mosquito vectors of dengue, chikungunya and Zika?**	
Absolutely not good	1.25 (5)
Not good	7.00 (28)
Moderate	29.75 (119)
Good	29.00 (116)
Very good	23.75 (95)
Do not know/ Do not answer	9.25 (37)
**Are you interested in implementing new technologies or methods to reduce the mosquito vectors of dengue, chikungunya and Zika in your household or community?**	
Extremely interested	24.50 (98)
Interested	38.25 (153)
Not sure	21.00 (84)
Not interested	1.25 (5)
Strongly not interested	0.50 (2)
Do not know/ Do not answer	14.50 (58)
**What factors influence your decision to adopt new technologies or methods to reduce dengue, chikungunya, and Zika in your homes or communities? (Multiple answers)**	
Price and cost effectiveness	14.00 (56)
Effectiveness	18.50 (74)
Human safety	24.25 (97)
Animal safety	2.75 (11)
Environmental safety	13.00 (52)

Characteristics	% (N = 400)
Others	0.25 (1)
Do not know/ Do not answer	27.25 (109)
