# Supplementary material for: Knowledge, attitude, and practice (KAP), and acceptance and willingness to pay (WTP) for mosquito-borne diseases control through sterile mosquito release in Bangkok, Thailand
Source: PLoS Negl Trop Dis. 2025 Jul 28;19(7):e0011935. doi: 10.1371/journal.pntd.0011935 (PMC12303319; doi:10.1371/journal.pntd.0011935)
Supplement: S7 Table — (PDF) [file pntd.0011935.s007.pdf]

S7 Table. Willingness to pay (WTP) for the application of sterile mosquitoes to reduce mosquito vectors of dengue, chikungunya, and Zika of the surveyed participants living in Bangkok, Thailand.

Characteristics	% (N = 400)	
**Which of the following methods will you choose if the cost is at your own expense?**		
Sterile mosquitoes (1)[a]	10.75 (43)	
Sterile mosquitoes (2)[b]	17.50 (70)	
Do not select both	52.00 (208)	
Do not know/ Do not answer	19.75 (79)	
**If the sterile mosquitoes are sold in the market, are you willing to pay for them?**	**Sterile mosquitoes (1)**	**Sterile mosquitoes (2)**
Yes	12.00 (48)	12.00 (48)
No	32.75 (131)	39.50 (158)
Do not know/ Do not answer	55.25 (221)	48.50 (194)
**Are you willing to pay 5 THB[c] for one sterile mosquito?**		
Yes	9.25 (37)	9.50 (38)
No	29.00 (116)	29.75 (119)
Do not know/ Do not answer	61.75 (247)	60.75 (243)
**Are you willing to pay 10 THB one sterile mosquito?**		
Yes	7.50 (30)	7.25 (29)
No	29.75 (119)	27.25 (109)
Do not know/ Do not answer	62.75 (251)	65.50 (262)
**Are you willing to pay 15 THB one sterile mosquito?**		
Yes	6.50 (26)	6.50 (26)
No	28.75 (115)	28.50 (114)
Do not know/ Do not answer	64.75 (259)	65.00 (260)
**Are you willing to pay 2.5 THB one sterile mosquito?**		
Yes	7.00 (28)	6.50 (26)
No	29.50 (118)	29.50 (118)
Do not know/ Do not answer	63.50 (254)	64.00 (256)
**What is the maximum amount you are willing to pay for each sterile mosquito?**	9.50 (38) median (range) 1 THB (1-250 THB)	9.00 (36) median (range) 2 THB (1 – 200 THB)
Do not know/ Do not answer	90.50 (362)	91.00 (364)
**Please indicate the reason why you refuse to pay for sterile mosquitoes.**		
Want to get sterile mosquitoes free from the Government	14.75 (59)	37.75 (151)
Want to know more information or scientific evidence about sterile mosquitoes	3.50 (14)	13.00 (52)
Cannot afford to buy sterile mosquitoes	10.50 (42)	17.75 (71)
Need other measures to prevent and control dengue, chikungunya and Zika	6.50 (26)	9.75 (39)
Others	1.25 (5)	2.25 (9)
Do not know/ Do not answer	63.25 (253)	19.50 (78)

[a]sterile mosquitoes (1) means 200 sterile mosquitoes being release every week consecutively for two years;

[b] sterile mosquitoes (2) means 200 sterile mosquitoes being release every other week consecutively for two years

[c] 1 US$ = 34 THB at the time of the study
