# Supplementary material for: Knowledge, attitude, and practice (KAP), and acceptance and willingness to pay (WTP) for mosquito-borne diseases control through sterile mosquito release in Bangkok, Thailand
Source: PLoS Negl Trop Dis. 2025 Jul 28;19(7):e0011935. doi: 10.1371/journal.pntd.0011935 (PMC12303319; doi:10.1371/journal.pntd.0011935)
Supplement: S1 File — (DOCX) [file pntd.0011935.s008.docx]

**Household questionnaires on environmental/economic/social impacts and quality of life and community participation related to the application of sterile mosquitoes to reduce the incidences of Dengue / Chikungunya / Zika in Bangkok**

Survey Details *Note: Please fill out the information before starting the interview.*

Interview Team.................................................Interviewer................................................................

Interview Date............../............./.................

Interviewee ID …………......................................................................................................................

| **Section 1: General information about respondents** |
| --- |

Q1. Gender of the interviewee 1. Male 2. Female

Q2. Age .......................Years 97. Unknown 98. Not answer

Q3. Family Status

1. Head of family 2. Spouse 3. Son/daughter 4. Relatives

9. Others .................. 97. Unknown 98. Not answer

Q4. Your marital status

1. Single 2. Boyfriend / girlfriend 3. Married 4. Divorce

5. Widow 97. Unknown 98. Not answer

Q5. What is the highest level of education?

1. Uneducated 2. Primary School 1-6 3. Secondary School 1-3

4. Secondary School 4-6 5. Completion of Secondary School 6

6. Bachelor's degree or equivalent 7. Postgraduate

97. Unknown 98. Not answer

Q6. Current occupation

1. Laborer 2. Agriculture/farmer 3. Trading/merchant

4. Government/State Enterprise officer 5. Company employee 6. Housekeeper

7. Others, identify........................ 97. Unknown 98. Not answer

Q7. Current household income

1. 5,000 Baht or less 2. 5,001- 10,000 Baht 3. 10,001- 15,000 Baht

4. 15,001- 20,000 Baht 5. 20,001- 30,000 Baht 6. More than 30,000 Baht

97. Unknown 98. Not answer

Q8. Number of family members.......................................................(include interviewee)

97. Unknown 98. Not answer

Q9. Housing characteristics

1. Rental house 2. Government officials/State Enterprise residence 3. Own House

97. Unknown 98. Not answer

Q10. Types of accommodation

1. House with garden 2. One-story house with high basement

3. Two-story or more house 4. Commercial building

5. Apartment, dormitory 6. Row house

9. Others, specify.......................... 97. Unknown 98. Not answer

**Part 2: Control of mosquito vectors of Dengue**/ **Chikungunya/ Zika**

**General knowledge about Dengue / Chikungunya / Zika**

Place a cross (X) over the box that matches the interviewee's answer.

| **Question** | **Dengue** | **Chikungunya** | **Zika** |
| --- | --- | --- | --- |
| Q11. Have you heard about these diseases? | □ Yes □ Never  □ Unknown  □ Not answer | □ Yes □ Never  □ Unknown  □ Not answer | □ Yes □ Never  □ Unknown  □ Not answer |
| Q12. What are your sources of information about these diseases? | □ Radio  □ Television  □ Newspapers  □ Online media  □ Poster / Brochure  □ Health official / Village health volunteer  □ Neighbor /  acquaintance  □ Workplace / school  □ Unknown  □ Not answer | □ Radio  □ Television  □ Newspapers  □ Online media  □ Poster / Brochure  □ Health official / Village health volunteer  □ Neighbor /  acquaintance  □ Workplace / school  □ Unknown  □ Not answer | □ Radio  □ Television  □ Newspapers  □ Online media  □ Poster / Brochure  □ Health official / Village health volunteer  □ Neighbor /  acquaintance  □ Workplace / school  □ Unknown  □ Not answer |
| Q13. Do you think these diseases are severe? | □ Severe  □ Mild  □ Unknown  □ Not answer | □ Severe  □ Mild  □ Unknown  □ Not answer | □ Severe  □ Mild  □ Unknown  □ Not answer |
| Q14. What kind of mosquitoes carry these diseases? | □ *Aedes* spp.  □ *Culex* spp.  □ *Anopheles* spp.  □ All kinds of mosquitoes  □ Unknown  □ Not answer | □ *Aedes* spp.  □ *Culex* spp.  □ *Anopheles* spp.  □ All kinds of mosquitoes  □ Unknown  □ Not answer | □ *Aedes* spp.  □ *Culex* spp.  □ *Anopheles* spp.  □ All kinds of mosquitoes  □ Unknown  □ Not answer |
| Q15. Identify breeding sites of *Aedes* mosquitoes | □ Stagnant water  □ Flowing tides  □ Dirty places  □ Water containers  □ Garbage disposal area  □ Drainage pipe system  □ Unknown  □ Not answer | □ Stagnant water  □ Flowing tides  □ Dirty places  □ Water containers  □ Garbage disposal area  □ Drainage pipe system  □ Unknown  □ Not answer | □ Stagnant water  □ Flowing tides  □ Dirty places  □ Water containers  □ Garbage disposal area  □ Drainage pipe system  □ Unknown  □ Not answer |
| Q16. How are these diseases transmitted from person to person? | □ By mosquito bites  □ By touching each other  □ By water  □ By flies  □ By other animals  □ Unknown  □ Not answer | □ By mosquito bites  □ By touching each other  □ By water  □ By flies  □ By other animals  □ Unknown  □ Not answer | □ By mosquito bites  □ By touching each other  □ By water  □ By flies  □ By other animals  □ Unknown  □ Not answer |
| Q17. When do you think *Aedes* mosquitoes bite people? | □ Day  □ Night  □ Day / Night  □ Evening / Dusk  □ Unknown  □ Not answer | □ Day  □ Night  □ Day / Night  □ Evening / Dusk  □ Unknown  □ Not answer | □ Day  □ Night  □ Day / Night  □ Evening / Dusk  □ Unknown  □ Not answer |
| Q18. Have you ever suffered from these diseases? | □ Yes □ Never  □ Unknown  □ Not answer | □ Yes □ Never  □ Unknown  □ Not answer | □ Yes □ Never  □ Unknown  □ Not answer |
| Q19. Do you have any acquaintances who have suffered from these diseases? | □ Yes □ No  □ Unknown  □ Not answer | □ Yes □ No  □ Unknown  □ Not answer | □ Yes □ No  □ Unknown  □ Not answer |

**Control and eradication of mosquito vectors of Dengue / Chikungunya / Zika**

Place a cross (X) over the box that matches the interviewee's opinions.

| **Question** | **Interviewee's Opinion** |
| --- | --- |
| Q20. How do you get rid of the breeding sites for mosquito larvae in and around your home? | □ Put lids on all water containers tightly  □ Change water in the water container weekly  □ Waterlogging container disposal/ garbage disposal  □ Brush and scrub inside water containers  □ Release fish to consume larvae in waterlogged containers.  □ Put larvicides/chemicals in waterlogged containers.  □ Scoop mosquito larvae out of waterlogged containers  □ Did not do anything  □ Unknown  □ Not answer |
| Q21. What methods do you use to protect yourself and your family members from mosquito bites? | □ Sleeping under mosquito nets  □ Using mosquito repellent coils  □ Installation of mosquito screens  □ Use fan and mosquito repellent  □ Use mosquito shock machine / mosquito bat  □ Use insecticide-impregnated mosquito nets  □ Apply mosquito repellent lotion when entering forest / garden.  □ Wear long-sleeved shirts and long pants when entering forest / garden.  □ Did not do anything.  □ Other specify..............................  □ Unknown  □ Not answer |

**Attitude towards Dengue / Chikungunya / Zika**

Place a cross (X) over it in the attitude box that matches the interviewee's opinions.

| **Question** | **Attitude** | | | | | |
| --- | --- | --- | --- | --- | --- | --- |
|  | **Totally agree** | **Agree** | **Uncertain** | **Disagree** | **Strongly disagree** | **Unknown/ Not answer** |
| Q22. It is essential to keep the house and surrounding areas clean. |  |  |  |  |  |  |
| Q23. It is the right thing to do to empty and scrub water storage containers once a week. |  |  |  |  |  |  |
|  |  |  |  |  |  |  |
| Q24. It is difficult to eliminate breeding sites of the mosquito vectors of Dengue / Chikungunya / Zika viruses. |  |  |  |  |  |  |
|  |  |  |  |  |  |  |
| Q25. Households with Dengue / Chikungunya / Zika patients must cooperate to eliminate mosquito breeding sites. |  |  |  |  |  |  |
|  |  |  |  |  |  |  |
| Q26. Health officials play a critical role in preventing Dengue / Chikungunya / Zika at the community level. |  |  |  |  |  |  |
|  |  |  |  |  |  |  |
| Q27 Disposal of mosquito breeding sites is the sole responsibility of health officials. |  |  |  |  |  |  |
|  |  |  |  |  |  |  |
| Q28. Sleeping under mosquito nets can prevent Dengue / Chikungunya / Zika. |  |  |  |  |  |  |
| Q29. It could be life-threatening if you are sick with Dengue / Chikungunya / Zika and do not treat quickly. |  |  |  |  |  |  |
| Q30. The best method to prevent Dengue / Chikungunya / Zika is to avoid mosquito bites. |  |  |  |  |  |  |

**Practice of vector control measures for Dengue / Chikungunya / Zika**

Clarification: Place an X in the field of practice that matches the interviewee's actual practice with the following criteria:

- Always means interviewee has behaved regularly / at least once a week.
- Occasionally means interviewee has behaved occasionally.
- Never did means interviewee did not treat even once.
- Not Eligible means no containers/materials mentioned in the area of the interviewee's household

| **Questions** | **Practice** | | | **Not eligible** |
| --- | --- | --- | --- | --- |
|  | **Always** | **Occasionally** | **Never did** |  |
| Q31. Have you ever explored mosquito larvae in drinking water containers, cement basins in bathrooms / toilets, or other water storage containers? |  |  |  |  |
| Q32. If you find larvae in drinking water containers, cement basins in bathrooms / toilets or other water storage containers, have you ever: |  |  |  |  |
| 1. Remove larvae |  |  |  |  |
| (2) Add abate sand |  |  |  |  |
| (3) Clean water containers |  |  |  |  |
| Q33. Do you change water and wash flower vases, spotted betel vases, plant pot saucers weekly? |  |  |  |  |
| Q34. Do you change water or add vinegar or detergent or salt in pantry leg saucers weekly? |  |  |  |  |
| Q35. Have you ever surveyed water-holding wastes such as coconut shells, cans, tires, in your household area, and have you overturned, burned, landfilled, or destroyed them weekly? |  |  |  |  |
| Q36. Do you put guppy fishes in water containers such as jars or cement basins? |  |  |  |  |
| Q37. Do you use lids to cover all water jars filled with water? |  |  |  |  |
| Q38. Do you sleep in a mosquito net at night? |  |  |  |  |
| Q39. Do you use mosquito repellent coils to repel mosquitoes? |  |  |  |  |

**Part 3: General knowledge about sterilization of mosquitoes**

**Awareness of information about sterilization of mosquitoes**

Q40. Have you ever heard about sterilization of mosquitoes in order to reduce the mosquito vectors of Dengue / Chikungunya / Zika?

1. Yes 2. No

97. Unknown 98. Not answer

Q41. If yes, where did you receive information about the two-step sterilization of mosquitoes in order to reduce the mosquito vectors of Dengue / Chikungunya / Zika? (can answer more than one)

1. Local newspapers 2. Village broadcast tower

3. Municipal officer 4. Billboard

5. Leaflet/flyer 6. Others, specify................................

97. Unknown 98. Not answer

Q42. Do you think you would give an information about mosquito sterilization to your family / acquaintances / neighbors?

1. Yes 2. No 3. Not sure

97. Unknown 98. Not answer

**General knowledge about sterilization of mosquitoes**

Q43. What kinds of mosquitoes are used for sterilization?

1. All kinds of mosquitoes 2. *Aedes* spp. 3. *Culex* spp.

4. *Anopheles* spp. 9. Others, identify................................

97. Unknown 98. Not answer

Q44. Which of the following are the methods used to sterilize *Aedes* mosquitoes?

1. Low dose irradiation

2. Inject bacteria that resist Dengue / Chikungunya / Zika viruses into the mosquito.

3. Both 1 and 2 are correct.

97. Unknown 98. Not answer

Q45. What sex of *Aedes* mosquitoes is used for sterilization?

1. Males 2. Females 3. Both 1 and 2 are correct.

97. Unknown 98. Not answer

Q46 . What is the difference between *Aedes* males and females?

1. Males do not feed on blood, only nectar.

2. Females feed on blood, carrying dengue virus.

3. Males have thick and long antennae.

4. Females do not have thick and long antennae.

5. All of the above 6. More than one item, specified .............................

7. Not sure 97. Unknown 98. Not answer

Q47. How sterile mosquitoes are different from wild mosquitoes?

1. Sterile mosquitoes are able to resist germs.

2. Sterile mosquitoes are unable to mate with wild mosquitoes.

3. Sterile mosquitoes can mate with wild mosquitoes but cannot produce offspring

4. Both 1 and 3 are correct 5. All are correct 97. Unknown 98. Not answer

Q48. Which of the following is correct?

1. Sterile mosquitoes can produce offspring.

2. Sterile mosquitoes are only female.

3. Sterile mosquitoes are not able to produce offspring.

4. Sterile mosquitoes are only male.

5. Both 3 and 4 are correct 6. All are wrong

97. Unknown 98. Not answered

Q49. What are the benefits of sterile mosquitoes?

1. Help reduce mosquito populations in households/communities

2. Reduce the risk of Dengue / Chikungunya / Zika

3. Both 1 and 2 are correct. 4. All are wrong 97. Unknown 98. Not answer

Q50. How often to release sterile mosquitoes in order to reduce mosquito populations in nature?

1. Every week 2. Every two weeks 3. Every month

4. Not sure 97. Unknown 98. Not answer

**Attitude towards the application of sterile mosquitoes in order to** **reduce the mosquito vectors of Dengue / Chikungunya / Zika**

Q51. Do you think sterile mosquitoes are effective, practical and safe for human, animals and environment?

1. Strongly agree 2. Agree 3. Not sure

4. Disagree 5. Strongly disagree 98. Do not know / Do not answer

Q52. Do you think sterile mosquitoes can be used to reduce mosquito vectors of Dengue / Chikungunya / Zika?

1. Yes 2. No 3. Not sure 98. Do not know / Do not answer

Q53. Do you think an application of sterile mosquitoes is more useful than chemicals in order to reduce the mosquito vectors of Dengue / Chikungunya / Zika?

1. More useful 2. Equally 3. Less useful

4. Useless 5. Not sure 98. Do not know / Do not answer

Q54. If an application of sterile mosquitoes can reduce the mosquito vectors of Dengue / Chikungunya / Zika, would you like to have the sterile mosquitoes introduced into your household or community?

1. Desperately need 2. Need 3. Not sure 4. Not require

5. Absolutely not need 98. Do not know/ Do not answer

Q55. Could you please give rating on the application of sterile mosquitoes in order to reduce the mosquito vectors of Dengue / Chikungunya / Zika?

| 1 | 2 | 3 | 4 | 5 |
| --- | --- | --- | --- | --- |
| Absolutely not good | Not good | Moderate | Good | Very good |

Q56**.** Are you interested in implementing new technologies or methods to reduce the mosquito vectors of Dengue / Chikungunya / Zika in your household or community?

1. Extremely interested 2. Interested 3. Not sure

4. Not interested 5.Strongly not interested

98. Do not know / Do not answer

Q57. What factors influence your decision to adopt new technologies or methods to reduce the mosquito vectors of Dengue / Chikungunya / Zika in your household or community? (can answer more than one)

1. Price and cost effectiveness 2. Effectiveness 3. Human safety

4. Animal safety 5. Environmental safety 6. Others, specify...........................

98. Do not know/ Do not answer

**Interviewees’ willingness to pay for the application of sterile mosquitoes in order to** **reduce the mosquito vectors of Dengue / Chikungunya / Zika**

Q58. When you, or someone in your family, are bitten by mosquitoes, did you or your family members perform any of the actions described in this list?

| **Answer** | **You** | **Family members** |
| --- | --- | --- |
| 1. Home treatment (e.g., use home-based medications, creams, sprays, etc.) |  |  |
| 1. Buy products for your own use (e.g., medicines, creams, sprays, etc.) |  |  |
| 1. Go to see a doctor |  |  |
| 1. Go to see a specialist (e.g., dermatologist) |  |  |
| 1. Not seeing a doctor and doing nothing |  |  |
| 1. Others, please specify…………………………….... |  |  |

Q59. How much does it cost for you to treat or consult a doctor?

| **List** | **Amount (Baht)** |
| --- | --- |
| 1. Expenses for purchasing products for your own use (e.g., medicines, creams, sprays, etc.) |  |
| 1. Expenses for seeing doctor / nurse |  |
| 1. Expenses for visiting a specialized doctor (e.g., dermatologist) |  |
| 1. Other expenses, please specify ............................ |  |

Q60. How much does your family incur for controlling mosquito vectors according to the list below?

| **List** | **Amount (Baht)** |
| --- | --- |
| 1. Repair leaking faucet and/or outdoor water pipe |  |
| 2. Repair window and/or door screen |  |
| 3. Add abate sand or larvicide in water containers inside and outside your household |  |
| 4. Use insect trap and mosquito trap |  |
| 5. Use mosquito repellent spray |  |
| 6. Use mosquito repellent cream or mosquito repellent candle |  |
| 7. Others, please specify ............................................... |  |

Q61. Imagine the following scenario and think of which methods you would use in that situation:

If the release of sterile mosquitoes is approved by the governmental agency in Thailand in order to reduce natural populations of *Aedes* mosquitoes and to reduce the spread of Dengue, Chikungunya and Zika, and the cost will be at your own expense. Which of the following methods will you choose?

| **Sterile mosquitoes (1)** | **Sterile mosquitoes (2)** |
| --- | --- |
| - Release approximately **200** sterile mosquitoes around your home **every week** for two consecutive years | - Release approximately **200** sterile mosquitoes around your home **every other** **week** for two consecutive years |
| - Reduce the number of *Aedes* mosquitoes in nature for 1-2 years | - Reduce the number of *Aedes* mosquitoes in nature for 1-2 years |
| - Reduce the use of pesticides | - Reduce the use of pesticides |
| - No pollution in the environment | - No pollution in the environment |
| - Use sterile mosquitoes (1) along with other methods of controlling mosquito larvae, such as eliminating mosquito breeding sites, using abate sand, using guppy fish, etc.) | - Use sterile mosquitoes (2) along with other methods of controlling mosquito larvae, such as eliminating mosquito breeding sites, using abate sand, using guppy fish, etc.) |

1. Sterile mosquito (1) 2. Sterile mosquito (2) 3. Do not select both (go to Q75)

| **Sterile mosquitoes (1)** | **Sterile mosquitoes (2)** |
| --- | --- |
| Q62. If sterile mosquitoes (1) are sold in the market, are you willing to pay for sterile mosquitoes (1)?  1. Yes 🡪 Go to Q63  2. No 🡪 Go to Q67 | Q69. If sterile mosquitoes (2) are sold in the market, are you willing to pay for sterile mosquitoes (2)?  1. Yes 🡪 Go to Q70  2. No 🡪 Go to Q75 |
| Q63. Are you willing to pay 5 baht for a sterile mosquito (1)?  1. Yes 🡪Go to Q64  2. No 🡪Go to Q66 | Q70. Are you willing to pay 5 baht for a sterile mosquito (2)?  1. Yes 🡪Go to Q71  2. No 🡪Go to Q73 |
| Q64. Are you willing to pay 10 THB for a sterile mosquito (1)?  1. Yes 🡪Go to Q65  2. No 🡪Go to Q69 | Q71. Are you willing to pay 10 THB for a sterile mosquito (2)?  1. Yes 🡪Go to Q72  2. No 🡪Go to Q74 |
| Q65. Are you willing to pay 15 THB for a sterile mosquito (1)?  1. Yes 🡪Go to Q67  2. No 🡪Go to Q68 | Q72. Are you willing to pay 15 THB for a sterile mosquito (2)?  1. Yes 🡪Go to Q74  2. No 🡪Go to Q75 |
| Q66. Are you willing to pay 2.50 THB for a sterile mosquito (1)?  1. Yes 🡪Go to Q67  2. No 🡪Go to Q68 | Q73. Are you willing to pay 2.50 THB for a sterile mosquito (2)?  1. Yes 🡪Go to Q74  2. No 🡪Go to Q75 |
| Q67. What is the maximum amount that you are willing to pay for each sterile mosquito (1)?  ___________________________ Baht | Q74. What is the maximum amount that you are willing to pay for each sterile mosquito (2)?  ________________________Baht |
| Q68. Please indicate the reason why you refuse to pay for sterile mosquitoes (1).   1. Want to get sterile mosquitoes free from the Government 2. Want to know more information or scientific evidence about sterile mosquitoes 3. Cannot afford to buy sterile mosquitoes   4. Need other measures to prevent and control of Dengue / Chikungunya / Zika    5. Others, please specify .................................... | Q75. Please indicate the reason why you refuse to pay for sterile mosquitoes (2).   1. Want to get sterile mosquitoes free from the Government 2. Want to know more information or scientific evidence about sterile mosquitoes 3. Cannot afford to buy sterile mosquitoes   4. Need other measures to prevent and control of Dengue / Chikungunya / Zika  5. Others, please specify ..................................... |

**Section 4: Impacts on environment, economic, social and quality of life**

**Impacts on environment, economic, social and quality of life** **of** **participants from** t**he** **release of sterile male mosquitoes** **in the study area**.

Place a cross (X) in the attitude box that matches the interviewee's opinions.

| **Question** | **Attitude** | | | | | |
| --- | --- | --- | --- | --- | --- | --- |
|  | **Totally agree** | **Agree** | **Uncertain** | **Disagree** | **Strongly disagree** | **Do not know / Do not answer** |
| **Environmental Impact** |  |  |  |  |  |  |
| Q76. Breeding sites for mosquito larvae are eliminated in household / community |  |  |  |  |  |  |
| Q77. Better waste management in household / community. Being waste-free or good hygienic communities |  |  |  |  |  |  |
| Q78. Cleaner household / community and pleasant environment |  |  |  |  |  |  |
| Q79. Can make use of mosquito catching equipment / tools in household / community |  |  |  |  |  |  |
| Q80. Number of mosquitoes in household / community decreases |  |  |  |  |  |  |
| Q81. Reduce the use of chemicals to control mosquitoes. Reduce residual pollution resulting from the use of chemicals in the environment |  |  |  |  |  |  |
| Q82. Household / community is free from Dengue / Chikungunya / Zika |  |  |  |  |  |  |
| **Economic impact** |  |  |  |  |  |  |
| Q83. Reduce household / community expense in purchasing mosquito repellent / anti-mosquito equipment |  |  |  |  |  |  |
| Q84. Reduce the cost of medical treatment because there is no risk of getting sick with Dengue / Chikungunya / Zika |  |  |  |  |  |  |
| Q85. No loss of income caused by the absence from work due to being sick with Dengue / Chikungunya / Zika |  |  |  |  |  |  |
| Q86. More saving from not having to pay for medical expense due to illness and not having to pay for mosquito repellent / anti-mosquito equipment |  |  |  |  |  |  |
| Q87. More income from being volunteer to collect mosquito samples in the area |  |  |  |  |  |  |
| Q88. Community shops have more income as researcher come to do research in the area |  |  |  |  |  |  |
| **Social Impact** |  |  |  |  |  |  |
| Q89. You discussed, consulted and taken responsibility about the elimination of mosquito breeding sites and mosquito eradication with your family members. |  |  |  |  |  |  |
| Q90. You get along well with your neighbors / people in the community because you participate in community activities. |  |  |  |  |  |  |
| Q91 You had more opportunity to talk, meet and discuss with neighbors / people in the community about mosquito control activities. |  |  |  |  |  |  |
| Q92. You receive health support / care / promotion from community volunteers / health officials. |  |  |  |  |  |  |
| Q93. You are ready to assist your neighbors / community in eliminating mosquito breeding sites and getting rid of mosquitoes. |  |  |  |  |  |  |
| Q94. After release of sterile mosquitoes, you and people within the community have a good relationship with each other. |  |  |  |  |  |  |
| Q95. You are satisfied with the living conditions after conducting the research project in the area. |  |  |  |  |  |  |
| Q96. You are better aware of various information from printed media, radio, television and community broadcast channel. |  |  |  |  |  |  |
| **Impact on the quality of life** |  |  |  |  |  |  |
| Q97. Do you feel safe and satisfied with your life because you are not afraid of getting sick with mosquito-borne diseases? |  |  |  |  |  |  |
| Q98. Are you in a good mood and have good mental health because of no stress due to good health, no illness? |  |  |  |  |  |  |
| Q99. Do you feel self-valued because you have contributed to reducing mosquito breeding sites / eradicating mosquitoes in your household / community? |  |  |  |  |  |  |
| Q100. You have the opportunity to take a break from the stress of the unhealthy environment. |  |  |  |  |  |  |
| Q101. Do you feel that you and your community have benefits from the release of sterile mosquitoes? |  |  |  |  |  |  |

**Section 5: Community engagement and project participation satisfaction**

***The questions in this section are for asking interviewees' opinions only after participating in the research project and releasing sterile male mosquitoes.***

**Community involvement in participating in the two-step mosquito sterilization project**

Place a cross (X) on a satisfaction scale that matches the interviewee's opinions.

| **Question** | **Satisfaction level** | | | | | | |
| --- | --- | --- | --- | --- | --- | --- | --- |
|  | **Very satisfied** | **Moderately satisfied** | **Less satisfied** | **Impartial** | **Not satisfied** | **Unknown** | **Not answer** |
| Q102. There is hearing / opinion / suggestion session from people in the community before starting a research project. |  |  |  |  |  |  |  |
| Q103. People in the community are involved in planning implementation of the research project. |  |  |  |  |  |  |  |
| Q104. People in the community can make suggestions to change the methods of conducting research projects to suit the community. |  |  |  |  |  |  |  |
| Q105. People in the community can participate in the research project if they are interested. |  |  |  |  |  |  |  |
| Q106. People in the community can ask questions about implementation of the research project throughout the project period. |  |  |  |  |  |  |  |
| Q107. People in the community can monitor progress of the research project on a regular basis. |  |  |  |  |  |  |  |
| Q108. People in the community can complain about adverse events resulting from implementation of the research project. |  |  |  |  |  |  |  |
| Q109. Community members / community leaders may cancel the research project at any time when there are acceptable grounds. |  |  |  |  |  |  |  |

**Satisfaction in participating in the two-step mosquito sterilization project.**

Place a cross (X) on a satisfaction scale that matches the interviewee's opinions.

| **Question** | **Satisfaction level** | | | | | | |
| --- | --- | --- | --- | --- | --- | --- | --- |
|  | **Very satisfied** | **Moderately satisfied** | **Less satisfied** | **Impartial** | **Not satisfied** | **Unknown** | **Not answer** |
| Q110. Details/procedures of the research project are clarified for easy understanding by the public. |  |  |  |  |  |  |  |
| Q111. The public receives full information about the pros / cons of the research project. |  |  |  |  |  |  |  |
| Q112. Results of the research project are regularly publicized / disseminated. |  |  |  |  |  |  |  |
| Q113. Appropriate knowledge promotion activities are organized. |  |  |  |  |  |  |  |
| Q114. Researchers can be contacted conveniently / quickly in the case of adverse events resulting from implementation of the research project. |  |  |  |  |  |  |  |
| Q115. The duration of the research project is appropriate. |  |  |  |  |  |  |  |
| Q116. The research project focuses on the interests of people in the area. |  |  |  |  |  |  |  |
| Q117. Releasing sterile mosquitoes can reduce the number of mosquitoes in your household / community. |  |  |  |  |  |  |  |
| Q118. Releasing sterile mosquitoes can reduce the rate of illness from Dengue / Chikungunya / Zika. |  |  |  |  |  |  |  |
| Q119. Releasing sterile mosquitoes can reduce the use of chemicals that used for controlling mosquitoes. |  |  |  |  |  |  |  |
| Q120. Releasing sterile mosquitoes can reduce the burden of medical expenses due to illness. |  |  |  |  |  |  |  |
| Q121. Releasing sterile mosquitoes in the community causes annoyance / stress / discomfort / intrusive. |  |  |  |  |  |  |  |
| Q122. There are concerns that the release of sterile male mosquitoes will increase the number of mosquitoes in household / community. |  |  |  |  |  |  |  |
| Q123. There are worries/uncertainties that releasing sterile mosquitoes will introduce new species of mosquitoes into household / community. |  |  |  |  |  |  |  |
| Q124. Release sterile mosquitoes may decrease mosquitoes in nature and, as a result, may cause nature imbalance. |  |  |  |  |  |  |  |
| Q125. There is satisfaction with implementation of the research project in the community. |  |  |  |  |  |  |  |
| Q126. If similar research projects are carried out in the community, you feel positive to cooperate and participate in those research projects. |  |  |  |  |  |  |  |

More suggestions

………………………………………………………………………………………………………………………

………………………………………………………………………………………………………………………

………………………………………………………………………………………………………………………

………………………………………………………………………………………………………………………

………………………………………………………………………………………………………………………
